# Empirically validated theoretical analysis of visual-spatial perception under change of nervous system arousal

**DOI:** 10.3389/fncom.2023.1136985

**Published:** 2023-05-12

**Authors:** Pratik Purohit, Prasun Dutta, Prasun K. Roy

**Affiliations:** ^1^School of Biomedical Engineering, Indian Institute of Technology (BHU), Varanasi, India; ^2^Department of Physics, Indian Institute of Technology (BHU), Varanasi, India; ^3^Department of Life Sciences, Shiv Nadar University (SNU), Greater Noida, India

**Keywords:** spatial perception, metric tensor, Hill equation, grid cell, visual cortex, psilocybin, autonomic nervous system, diffusion tensor imaging

## Abstract

**Introduction:**

Visual-spatial perception is a process for extracting the spatial relationship between objects in the environment. The changes in visual-spatial perception due to factors such as the activity of the sympathetic nervous system (hyperactivation) or parasympathetic nervous system (hypoactivation) can affect the internal representation of the external visual-spatial world. We formulated a quantitative model of the modulation of visual-perceptual space under action by hyperactivation or hypoactivation-inducing neuromodulating agents. We showed a Hill equation based relationship between neuromodulator agent concentration and alteration of visual-spatial perception utilizing the metric tensor to quantify the visual space.

**Methods:**

We computed the dynamics of the psilocybin (hyperactivation-inducing agent) and chlorpromazine (hypoactivation-inducing agent) in brain tissue. Then, we validated our quantitative model by analyzing the findings of different independent behavioral studies where subjects were assessed for alterations in visual-spatial perception under the action of psilocybin and under chlorpromazine. To validate the neuronal correlates, we simulated the effect of the neuromodulating agent on the computational model of the grid-cell network, and also performed diffusion MRI-based tractography to find the neural tracts between the cortical areas involved: V2 and the entorhinal cortex.

**Results:**

We applied our computational model to an experiment (where perceptual alterations were measured under psilocybin) and found that for *n* (Hill-coefficient) = 14.8 and *k* = 1.39, the theoretical prediction followed experimental observations very well (χ2 test robustly satisfied, *p* > 0.99). We predicted the outcome of another psilocybin-based experiment using these values (*n* = 14.8 and *k* = 1.39), whereby our prediction and experimental outcomes were well corroborated. Furthermore, we found that also under hypoactivation (chlorpromazine), the modulation of the visual-spatial perception follows our model. Moreover, we found neural tracts between the area V2 and entorhinal cortex, thus providing a possible brain network responsible for encoding visual-spatial perception. Thence, we simulated the altered grid-cell network activity, which was also found to follow the Hill equation.

**Conclusion:**

We developed a computational model of visuospatial perceptual alterations under altered neural sympathetic/parasympathetic tone. We validated our model using analysis of behavioral studies, neuroimaging assessment, and neurocomputational evaluation. Our quantitative approach may be probed as a potential behavioral screening and monitoring methodology in neuropsychology to analyze perceptual misjudgment and mishaps by highly stressed workers.

## 1. Introduction

The organism or the human body is situated in the surroundings. The sensory system in the human body conveys information about the surroundings to the nervous system. The brain organizes and interprets this information in the collective process, termed as perception. Perception forms an internal map of the outside world and significantly affects everyday life. For example, red color is physically a particular wavelength of electromagnetic radiation but perceptually mapped as a visual property. Its presence can psychologically affect decision-making (Elliot et al., 2007). Perception of an external event is a complex process and depends on various factors apart from sensory information. For example, in the case of the Ponzo illusion, the perceived length of the two physically equal straight lines is unequal because of the contextual information (Ponzo, 1911). Various sensory illusions, such as tactile, auditory, olfactory, and visual illusions, indicate that the perceived environment may differ from reality due to the particular organization of the sensory information (Ponzo, 1911; Eagleman, 2001; Kalderon, 2011; Lederman and Jones, 2011; Stevenson, 2011). However, even if the sensory information is the same, a person can perceive sensory illusions differently if the biochemical activity in the brain is modulated due to any endogenous or exogenous factor (Van Loon et al., 2013; Costa et al., 2021). One such factor is the arousal or activation of the autonomic nervous system, which influences biochemical activity and thus can modulate perception of the external environment.

The autonomic nervous system regulates the physiological response to immediate stress by the “fight-or-flight” response, referred to as hyperactivation. Reciprocally, a “rest-and-digest” state facilitates relaxation and recovery, referred to as hypoactivation (McCorry, 2007). Hyperactivation and hypoactivation may not necessarily occur in response to an external hazard, but they can occur due to other factors such as neurochemical or neuromodulator action or sensory stimuli. Hyperactivation and hypoactivation are associated with the actuation of the sympathetic and parasympathetic nervous systems, respectively. Indeed, hyperactivation and hypoactivation of the autonomic nervous system cause perceptual variations and distortions in the visual perspective, handwriting area, finger tapping rate, taste threshold, brightness sensitivity, etc (Fischer, 1971). The brain forms a perceptual map of the environment based on sensory information. However, even without changes in incoming sensory information, variations in the perceptual experiences can be due to the modulation of cerebral activity while the neurophysiological activation varies. Therefore, the differences in the nervous system arousal levels should correlate with the inter-individual variability of the perception of the same event.

Several factors can induce the alteration of the activation level. Nevertheless, biochemical alterations of arousal due to an oral dose of a neuromodulator agent in an experimental setting are more convenient for mathematical modeling of the activation level. Examples of hyperactivation state-inducing neuromodulating agents include tryptamine-derivatives, glycolate, psilocybin, and adrenochrome. Conversely, hypoactivation state-inducing neuromodulating agents encompass phenothiazine-derivatives, dextomid, chlorpromazine, and alimemazine. The two groups of neuromodulating agents act by activating the sympathetic and parasympathetic nervous systems, respectively. When administered, such agents cross the blood-brain barrier, diffuse in the brain’s extracellular space, and produce pharmacological activity (Pardridge, 2012).

Autonomic nervous system activity has been the focus of various studies which observed the corresponding impact on visual perception. For instance, research has indicated that the Mueller-Lyer illusion, a well-known visual illusion, was modified by LSD-induced sympathetic nervous system activity (Edwards and Cohen, 1961; Olbrich et al., 2021). Another study found an association between psilocybin-induced sympathetic activity and change in the handwriting area, an indication of alteration in visual-spatial perception (Fischer et al., 1969b). Moreover, alterations in brightness preference (Fischer et al., 1969a) and visual-motion perception (Carter et al., 2004) were observed due to the activity of the sympathetic nervous system modulated by the psilocybin action. Similarly, the activity of the parasympathetic nervous system also affects visual perception. The chlorpromazine-induced parasympathetic nervous system alters the spatial distortion threshold, a direct manifestation of the corresponding alteration in visual perception (Hill and Fischer, 1971). Despite the experimental observations of the changes in visual perception with autonomic nervous system activity, a quantitative, computational, and theoretical model of the underlying phenomenon is still lacking. To date, no significant attempts have been made to provide a quantitative explanation of how neurochemical-induced changes in perception impact the spatial mapping of the visual field from physical to perceptual space. Therefore, there remains a need to develop such a model, as it could provide insight into the underlying mechanisms and help to further our understanding of the complexities of human visual perception.

Several experimental studies have measured the perceptual alterations associated with different sensory modalities under the influence of hyperactivation and hypoactivation agents (Fischer et al., 1969b; Hill et al., 1969; Wittmann et al., 2007). Thus, the hyperactivation and hypoactivation states alter the neurochemical milieu across the brain and impact perception associated with all sensory modalities.

In this paper, we will focus specifically on the visual system and examine how hyperactivation and hypoactivation situations induced by neuromodulating agents can alter visual spatial perception. To the best of our knowledge, this paper presents the first novel quantitative neurophysiological analysis and empirically validated computational model of perceptual alterations and distortions with respect to the dynamics of hyperactivation and hypoactivation inducing agents in brain tissue. To achieve this, we have formulated the kinetics-based action of neuromodulating agents and developed a geometrical model to describe and understand the corresponding changes in visual spatial perception. We have validated our computational formulation by analyzing findings from behavioral studies where subjects were assessed to estimate visuospatial perceptual alteration and distortion under the action of both hyperactivation and hypoactivation-inducing agents. Furthermore, we will explore the neuronal basis that can manifest as alterations in visual-spatial perception by simulating a drug action on a computational model of the a grid cell network. Additionally, we will use MRI tractography to identify the neural tracts that may be part of the brain network responsible for visual-spatial perception.

We have organized our paper as follows:

(i) In section “2. Theoretical analysis and mathematical modeling,” we have formulated the mathematical model to quantify the modulation of the visual spatial perception, due to change of arousal levels by pharmacological agents.
  We have divided section “2. Theoretical analysis and mathematical modeling” into two sub-sections:
  • Section “2.1. Visual-spatial perception modulated by pharmacological agents”: Mathematical framework for showing the relationship between the visual-spatial perception (shown by metric tensor) and drug concentration.
  • Section “2.2. Neural correlates of drug-induced modulation of visual-spatial perception”: Neuronal model of the representation of the visual space by grid cell network under the action of the neuromodulating drug.
(ii) In section “3. Materials and methods,” we have described the methodology used for experimental investigations.
  We have divided section “3. Materials and methods” into three sub-sections:
  • Section “3.1. Drug dynamics in the brain.”
  • Section “3.2. Diffusion MRI tractography.”
  • Section “3.3. Neuronal network model of grid cells.”
(iii) Section “4. Results” shows the results obtained after applying our theoretical formulation (see section “2. Theoretical analysis and mathematical modeling”) using the methodology described in section “3. Materials and methods.”
  We have divided the section “4. Results” into three sub-sections:
  • Section “4.1. Validation based on independent experiments”: Empirical study of our model using different behavioral studies.
  • Section “4.2. Anatomical connectivity between the entorhinal cortex and visual cortex (area V2)”: Corroboration of our approach by neuroanatomical tract study.
  • Section “4.3. Neuronal network basis of drug-induced modulation of spatial perception”: Simulating the effect of the neuropharmacological agents on the spatial mapping process by grid cell network.
(iv) Discussing the key results, their implications, and future prospects are in six sub-sections of section “5. Discussion.”

## 2. Theoretical analysis and mathematical modeling

### 2.1. Visual-spatial perception modulated by pharmacological agents

#### 2.1.1. The modulated geometry of the perceived visual space

The visual system senses optical information and sends neural signals to the brain, which constructs a perceptual map of the external world, called visual space. Visual space encodes dynamic relationships among objects and is the subjective counterpart of physical space. Despite the external world seeming Euclidean to us, studies showed that the geometry of visual space is non-euclidean (Luneburg, 1947; Indow, 1979; Fernandez and Farell, 2009; Koenderink et al., 2016). Since the activation of the autonomic nervous system elicits the modulation of visual perception (Fischer et al., 1969a,b, 1970; Hill et al., 1969; Fischer, 1971; Hill and Fischer, 1971), it can be taken that the visual space will remain non-euclidean under the hyper or hypoactivation state. Therefore, hypoactivation and hyperactivation induced alterations in visual perception can be represented by variations in the non-euclidean geometry of the visual space.

We will now explore the mathematical formulation that can represent the geometry of the visual space. The distance (ds) between two infinitesimal closed points whose cartesian coordinates are (x_1_, y_1_) and (x_1_+dx, y_1_+dy) on a two-dimensional flat or euclidean space can be calculated using the Pythagoras theorem, as shown in Equation (1).


(1) $$d\,s^{2}=d\,x^{2}+d\,y^{2}$$

Rewriting Equation (1) in matrix form:


$$d\,s^{2}=\left[\begin{array}{cc} d\,x & d\,y \end{array}\right]\,\left[\begin{array}{cc} 1 & 0\\ 0 & 1 \end{array}\right]\,\left[\begin{array}{c} d\,x\\ d\,y \end{array}\right]$$

(2) $$d\,s^{2}=\left[\begin{array}{cc} d\,x & d\,y \end{array}\right]\,g\,\left[\begin{array}{c} d\,x\\ d\,y \end{array}\right]$$

However, if two points are located on the curved or non-euclidean space, the Pythagoras theorem does not hold. The distance (ds) between two infinitesimal points can be calculated for the non-euclidean spaces using Equation (3).


(3) $$d\,s^{2}=\alpha .d\,x^{2}+\beta .d\,y^{2}+\theta .d\,x\,d\,y+\Phi .d\,y\,d\,x$$

Rewriting Equation (3) in matrix form


$$d\,s^{2}=\left[\begin{array}{cc} d\,x & d\,y \end{array}\right]\,\left[\begin{array}{cc} \alpha & \Phi \\ \theta & \beta \end{array}\right]\,\left[\begin{array}{c} d\,x\\ d\,y \end{array}\right]$$

(4) $$d\,s^{2}=\left[\begin{array}{cc} d\,x & d\,y \end{array}\right]\,g\,\left[\begin{array}{c} d\,x\\ d\,y \end{array}\right]$$

**FIGURE 1:**
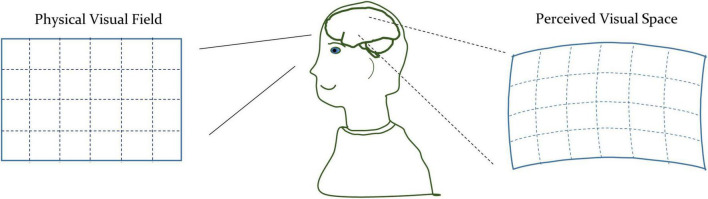
Conceptual illustration of the physical space and perceived visual space under the influence of th drug-induced activation of the autonomic nervous system.

A comparison of Eqs. 2, 4 shows the difference between the non-euclidean and euclidean spaces. In euclidean space, the diagonal components of matrix g are unity, and other components are zero. While in the non-euclidean space, the components of the matrix *g* can be arbitrary, depending upon the underlying geometry. The standard mathematical term for the matrix *g* is the metric tensor. The metric tensor (g) in Equation (4) is generalizable to the n-dimensional space. In addition to defining the way to measure distances, the metric tensor also expresses the geometrical characteristics of the arbitrary space. Therefore, we will use the metric tensor as an indicator of the underlying geometrical variations of the visual space due to the modulation of the visual perception. We illustrated a representative perceived geometry of the physical visual field in Figure 1.

As the pharmacological agent is administered, its concentration will gradually increase in the brain’s extracellular space after crossing the blood-brain barrier (Pardridge, 2012; Daneman and Prat, 2015), reach a peak, and then decline as the agent is metabolized and excreted via the kidney (Anders, 1980). Since the metric tensor of the perceived visual space (without any pharmacologically induced nervous system activation) may vary individually due to current internal and external factors, we will consider the changes in the metric tensor components to quantify the effect of the nervous system activation on visual perception. Several experimental investigations have suggested that visual space may be isotropic (Yartsev and Ulanovsky, 2013; Kim et al., 2017; Kim and Maguire, 2018). Thus, off-diagonal components of the metric tensor may not change significantly due to the alterations of the visual perception, and only diagonal components should be sufficient to capture the dynamic modulatory influence of nervous system activation on visual perception.

Furthermore, the type of nervous system activation affects visual perception oppositely (Fischer, 1971), which can be taken as the opposite changes in the metric tensor components. To paraphrase, hyperactivation can increase the value of the metric tensor components, while hypoactivation can decrease the value of the metric tensor components. An individual’s pharmacological tolerance or sensitivity can affect the amount of the drug needed to induce the perceptual alterations, personalized weber constant (P) represent this sensitivity. The lower value of the weber constant indicates that less drug is required to elicit perceptual alterations, i.e., the person has higher sensitivity to the agent.

Hence, we may write the change in the metric tensor in spherical coordinates (r, θ, Φ) as follows in Equation (5):


(5) $$△\,\text{g}=\left[\begin{array}{ccc} \frac{\mu \,{\text{Mg}}_{\text{rr}}^{\text{max}}}{\text{P}} & 0 & 0\\ 0 & {\text{r}}^{2}\,\frac{\mu \,{\text{Mg}}_{\theta \,\theta }^{\text{max}}}{\text{P}} & 0\\ 0 & 0 & {\text{r}}^{2}\,\cos^{2}\theta \,\frac{\mu \,{\text{Mg}}_{\Phi \,\Phi }^{\text{max}}}{\text{P}} \end{array}\right]$$

where μ = arousal coefficient (μ = 1 for hyper-activation and μ = −1 for hypo-activation inducing drugs).

P = Personalized Weber constant.

*$g_{r\,r}^{m\,a\,x},g_{\theta \,\theta }^{m\,a\,x},g_{\Phi \,\Phi }^{m\,a\,x}$* Maximum possible deviation in metric tensor (g) components at *r* = 1 and θ = 0 (during the peak perceptual effect).

The metric tensor can also be calculated in any other coordinate system (Oeijord, 2005). In Equation (5), M is a modulation index representing the modulatory effect of drug molecules on the visual perception process. The numerical value of the modulation index (M) varies between 0 (no drug condition, △*g* = 0) and 1 (peak perceptual deviation, △*g*_*diagonal*_ = *$g_{r\,r}^{m\,a\,x},g_{\theta \,\theta }^{m\,a\,x},g_{\Phi \,\Phi }^{m\,a\,x}$* at *r* = 1 and θ = 0). The arousal coefficient (μ) denotes the type of nervous system activation, μ = 1 for hyperactivation and μ = −1 for hypoactivation. Note that, in this model, the parameter M can be taken out of the metric in Equation (5) as a multiplicating factor. Hence, change in the metric as response to the drug manifests as a diffeomorphic distortion induced by the parameter M. The change in the parameter M results due to the drug action, as will be shown subsequently.

In the following subsection “2.1.2. Modulation index,” we will derive the non-linear quantitative relationship between the drug concentration and modulation index (M).

#### 2.1.2. Modulation index

After diffusing into the extracellular space of the brain, the drug affects neuronal activities by affecting the neurochemical processes, and the subjects start to perceive changes in visual spatial perception, which is evident from the observations of the experimental studies (Fischer et al., 1969a,b, 1970; Hill et al., 1969; Fischer, 1971; Hill and Fischer, 1971). However, experimental studies could measure only perceptual properties/experiences, such as the handwriting area (Fischer et al., 1969b), which is a manifestation of the modulated geometry of the visual space. Since drug-tissue interaction in the brain is a chemical process, corresponding perceptual variations should correlate with the dynamics of this process. The pathways and mechanism of action of drug molecules vary and act differently in the brain depending on the drug’s chemical structure (van Rossum, 2011). However, irrespective of the drug’s chemical structure, drug molecules can affect the firing characteristics of the neurons and modulate the inter-neuronal signaling in the neuronal networks responsible for perceptual functions, albeit differently.

Next, we formulated a modulation index (M) whose value shows the level of alteration in the visual perception. The value of the M can vary from 0 to 1, where *M* = 1 denotes the peak perceptual response of the drug action, while *M* = 0 represents the normal condition. The mathematical form of the modulation index (M) is shown in Equation (6) [Please see Supplementary section S1 for the derivation of Equation (6)].


(6) $$M=\frac{1}{1+{\left(\frac{\text{k}}{C}\right)}^{n}}$$

Equation (6) is similar to the Hill equation, which is often used in biochemistry (Frank, 2013). Indeed, the occurrence of the Hill equation in our approach is not unexpected as drug-induced modulation of the visual-spatial perception is also a neurochemical process at the molecular level. Comparing the standard Hill equation and Equation (6) concludes that *n* is a Hill coefficient. Thus, the modulation index (M) is a function of the drug concentration (C), Hill coefficient (n), and k (half effective drug concentration) where the Hill coefficient (n) determines the rate of change of modulation index with changes in drug concentration, as represented in Supplementary Figure S1. In the following subsection “2.1.3. Drug dynamics in the brain,” we will formulate a mathematical dynamical model of the drug dynamics after ingesting a particular amount.

#### 2.1.3. Drug dynamics in the brain

Now, we will formulate a quantitative model of drug dynamics in the brain tissue. Drugs can induce either neurophysiological hyperactivation or hypoactivation. After the drug ingestion, the drugs cross the blood-brain barrier, whence the drug molecules diffuse into the extracellular space of the brain tissue (Pardridge, 2012; Daneman and Prat, 2015). Brain parenchyma is not a continuous space; therefore, it is not a free but rather a restricted diffusion. Therefore we are considering the brain as a porous medium (Gitis and Rothenberg, 2020) for the diffusion of drug molecules. The solid part of the porous medium is called the frame, and gapes between them as pores (Gitis and Rothenberg, 2020). Applying the concepts of the porous media in the case of the brain yield that the intracellular and extracellular spaces (Sun and Sun, 2021) are the frame and pores (Gitis and Rothenberg, 2020), respectively, as shown in Supplementary Figure S2. The intracellular space consists of the internal portion of the neuronal cells, while the extracellular space is the empty portion between neuronal cells filled with interstitial fluid (Sun and Sun, 2021).

Due to the porous structure of the diffusion space, the effective diffusivity of the drug molecules decreases by the factor of the square of the tortuosity (Lehner, 1979), where tortuosity (TS) is the ratio of the actual distance traced by the drug molecules and straight line distance (i.e., displacement). Using Fick’s second law (Macdonald, 1977) to represent the drug diffusion in the porous medium [Equation (7)]:


(7) $${\frac{\delta \,C}{\delta \,t}}_{d\,i\,f\,f\,u\,s\,o\,n}=\frac{D}{T\,S^{2}}\,\left(\frac{\partial^{2}C}{\partial x^{2}}+\frac{\partial^{2}C}{\partial y^{2}}+\frac{\partial^{2}C}{\partial z^{2}}\right)$$

where:

D = Diffusivity of the drug molecules in the extracellular space (cm^2^ per second).

TS = Tortuosity.

C = Drug concentration (moles per cm^3^).

x, y, z : axes of the three-dimensional cartesian coordinate system.

The porosity reduces the accessible volume available to the drug molecules by the factor of the volume fraction (α–the ratio of extracellular space volume and total volume) (Nicholson et al., 1979; Nicholson and Phillips, 1981). Next, drug molecules’ movement from the blood to the extracellular space through the blood-brain barrier depends on the transfer coefficient for the drug molecules from blood to the brain tissue (k_*po*_) and instantaneous drug concentration in the blood (C_*a*_) (Patlak and Fenstermacher, 1975). Another transfer coefficient for the drug molecules from the blood to the brain tissue (k_*pi*_) and instantaneous drug concentration in the brain (C) determines the dynamics of drug molecules crossing from the brain’s extracellular space to the vascular supply to the brain (blood) (Patlak and Fenstermacher, 1975).

Hence, we can represent the phenomenons mentioned above in mathematical form as follows [Equation (8)]:


(8) $${\frac{\delta \,\text{C}}{\delta \,\text{t}}}_{m\,o\,v\,e\,m\,e\,n\,t}=\frac{1}{\alpha }\,\left({\text{k}}_{\text{po}}\,{\text{C}}_{\text{a}}-{\text{k}}_{\text{pi}}\,C\right)$$

For our analysis, we are discounting the bulk flow of the drug molecules, as diffusion is the dominant process involved in the dynamics of the drug molecules (Ohata and Marmarou, 1992). Furthermore, we can take that the drug molecules are not diffusing from the extracellular space to the intracellular space. Using Eqs. (7, 8), we can now formulate a differential equation [Equation (9)] to represent drug dynamics in the brain where drug concentration (C) varies with time (t) and spatial location (x, y, z).


(9) $$\frac{\partial C}{\partial t}=\frac{D}{T\,S^{2}}\,\left(\frac{\partial^{2}C}{\partial x^{2}}+\frac{\partial^{2}C}{\partial y^{2}}+\frac{\partial^{2}C}{\partial z^{2}}\right)-\frac{\left(k_{p\,i}\,C-k_{p\,o}\,C_{a}\right)}{\alpha }$$

where:

C [= C(x, y, x, t)] = Drug concentration in the extracellular space (moles per cm^3^).

k_*pi*_ = Transfer coefficient for the drug molecules from brain tissue to the blood (per minute).

k_*po*_ = Transfer coefficient for the drug molecules from the blood to the brain tissue (ml per gram of tissue per minute).

C_*a*_ = Concentration of the drug in the plasma (moles per ml).

α = Volume fraction of the extracellular space with respect to the total volume.

### 2.2. Neural correlates of drug-induced modulation of visual-spatial perception

#### 2.2.1. Multi-modal sensory perception systems

In the previous sections, we have represented hyperactivation and hypoactivation induced alterations in the visual-spatial perception as a metric tensor of the perceived visual space whose components vary depending upon the type of the drug and the amount of the dose, as evident from Equation (5). The metric tensor defines the mathematical basis to compute the distances on the geometry of the perceived visual space. As per our formulation, the metric tensor components alter due to the modulatory action of the drug molecules.

Now we demarcate the neuronal procedures that may be utilized in the brain for the metric representation of the perceptual space related to the different perceptual modalities, such as visual space, auditory space, olfactory space, and sensorimotor representational space. Neuronal recordings from the entorhinal cortex of the freely moving rats led to finding the spatially modulated receptive field of the grid cells (Hafting et al., 2005). Grid cells in the entorhinal cortex encode position in the navigation space and correspond to the metric component of the navigational space (Moser et al., 2008; Dang et al., 2021). Grid cells exist in the entorhinal cortex of bats, monkeys, and humans (Yartsev et al., 2011; Killian et al., 2012; Jacobs et al., 2013). Apart from the entorhinal cortex, grid cells-like neural representations exist while navigating in the olfactory space, auditory space, and sensorimotor space, indicating a general nature of the grid cells to represent metric cognitive maps associated with different types of sensory modalities (Constantinescu et al., 2016; Horner et al., 2016; Aronov et al., 2017; Bao et al., 2019; Long and Zhang, 2021). Initially proposed by Tolman (1948), the brain represents and organizes the information or concepts and extracts the relationships between them from the cognitive map.

As we have delineated above, the grid cell-like neuronal representation is associated with various perceptual modalities. Similarly, in the case of visual space, activation of the grid cell in the entorhinal cortex is observed during (i) the head-fixed monkeys gazing at different images (Killian et al., 2012) and (ii) humans fixating on randomly appearing targets at different locations in the virtual visual environment (Nau et al., 2018). However, grid cells can also exist outside the entorhinal cortex, e.g., in the primary somatosensory cortex. Grid cell activity has been experimentally recorded in the primary somatosensory cortex while rats explored the given arena for the food pellets (Long and Zhang, 2021). In a different experiment, the grid cell activity in the medial entorhinal cortex was recorded in rats collecting randomly thrown food pellets in an enclosure (Hafting et al., 2005). In previous two experiments, the rats were doing the same task—*foraging for the food pellets*. But, grid cells were observed in the different brain regions (entorhinal cortex and primary somatosensory cortex). Therefore, we can infer that multiple simultaneous grid cell-based neural maps can exist during a particular task.

During navigation, grid cells in the entorhinal cortex can maintain their firing map independent of the environmental clues. However, path integration in grid cells integrates direction and speed information with firing positions (McNaughton et al., 2006). Considering the rats foraging for the food task, we can formulate that (i) the somatosensory system provides the necessary information for path integration to the grid cells in the entorhinal cortex and (ii) the somatosensory cortex organizes this path integration information in grid cells-like representation, thus constituting a local spatial map.

#### 2.2.2. Grid-cell based motif for mapping of visual space

We can now adapt our analysis to visual-spatial perception. Here, we consider two investigations: (i) the experimental observation of the grid cells in the entorhinal cortex during a visual task (Killian et al., 2012), and (ii) another experimental observation of the encoding of the spatial position in the primary visual cortex (Saleem et al., 2018). Accordingly, we may indicate that the grid cell-like activity should be present in the visual cortex, constituting a local visuospatial map. In the case of the visual system, grid cell like mapping is experimentally recorded in the area V2 of the visual cortex (Long et al., 2021). Indeed, we can posit this mapping because of the resemblance of functional cell population in the medial entorhinal cortex with (i) area V2 and with (ii) broader association cortices in the cerebral hemispheres (Moser et al., 2014).

Likewise, since the entorhinal plan can be the motif of broader cerebral cortical regions (Hawkins et al., 2019; Chen et al., 2022), then it can be construed that the local maps encoded by grid cells may be present in the other sensory modalities, and these maps may be linked to the entorhinal-hippocampal network for integrating sensory information with the prior information (memory). We represented our conceptual model in Supplementary Figure S3, involving the different sensory modalities. Furthermore, the grid cells are present in the orbitofrontal cortex, posterior cingulate cortex, ventromedial prefrontal cortex, and retrosplenial cortex (Constantinescu et al., 2016; Aronov et al., 2017). Hence, we can implicate that the grid cell like representation also encodes higher-level cognitive conceptual maps in various cortical systems in addition to the sensory-perceptual maps and these grid cells are linked to the hippocampal-entorhinal cortex network.

When applied to a specific sensory modality as the visual system, we can now indicate that this integrated network of the “local spatial map in the visual cortex” and the “entorhinal-hippocampal network” may be taken to represent the geometry of the perceived visual space.

**FIGURE 2:**
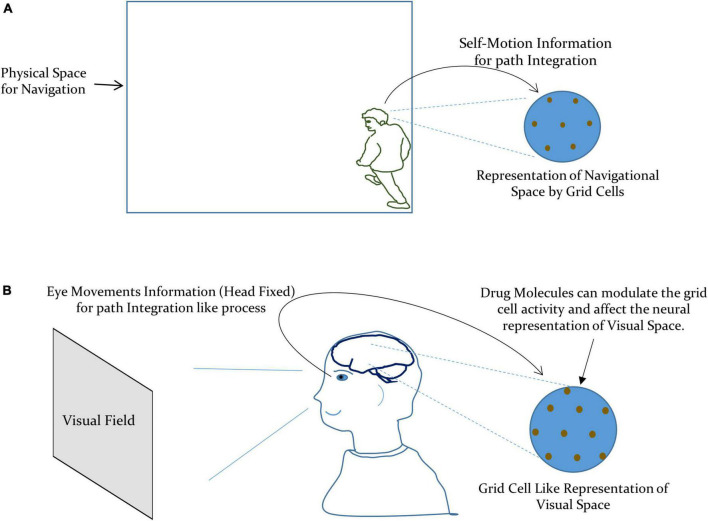
**(A)** Grid cells encode the navigational space by integrating the self-motion information for path integration. **(B)** Our formulation of the metric representation of the visual space by grid cells by integrating the information about the eye movements for path integration like process.

Thus, the modulation of the perceived visual space due to drug-induced activation of the autonomic nervous system can be taken as due to the alteration in the activity of: (i) the aforesaid integrated network of the entorhinal-hippocampal system and (ii) local spatial map at the visual cortex. This modulation may manifest as the change in the metric tensor components as a consequence of the modification in the grid cell representation of visual space at the neuronal level, under the effect of the drug. In the case of the navigational space, the grid cell encodes the external spatial location by integrating the self-motion information, as illustrated in Figure 2A (Etienne et al., 1996; Jeffery et al., 1997; McNaughton et al., 2006). However, in the case of the visual space, the eye movements (with the head fixed) can be taken as the primary information required for path integration like process in the grid cells. This is supported by the experimental observations where the grid cell representation emerged under the eye moving to explore the visual field (Killian et al., 2012; Nau et al., 2018). We have depicted our formulation in Figure 2B.

#### 2.2.3. Neuronal network model of drug-induced perceptual alteration

Firing patterns of different grid cells encode the necessary spatial information, and the firing field of a grid cell is characterized by three properties: grid scale, grid orientation, and grid spatial phase (Anton et al., 1970; Hafting et al., 2005; Aronov et al., 2017). Since a single grid cell has a periodic spatial field, it cannot encode a unique position. Nevertheless, the network of the grid cells [having different firing field characteristics and organized in different modules (Stensola et al., 2012)] can encode the unique positions. Thereby, we can formulate that the grid cell networks encode perceived space, consequently playing an essential role in the metric representation of the perceptual space of an individual.

It has been consistently shown that continuous attractor networks can model grid cell networks and firing patterns of the grid cells (Fuhs and Touretzky, 2006; McNaughton et al., 2006; Burak and Fiete, 2009; Couey et al., 2013). A continuous attractor network has a continuous set of states toward which the network evolve (Amit, 1989). The toroidal connectivity between the nodes in the continuous attractor network implements the periodic boundary for path integration (Samsonovich and McNaughton, 1997). Recently, the 3-D visualization of the experimentally obtained grid cell activity yielded a torus geometry where points on the torus correspond to the position in the physical space and thus validated the toroidal topology used in the theoretical models of the grid cell networks (Gardner et al., 2022; Hermansen et al., 2022).

## 3. Materials and methods

### 3.1. Drug dynamics in the brain

#### 3.1.1. Psilocybin

We obtained indicative parameters for psilocybin from various experimental studies to solve Equation (9). Tortuosity (TS) and volume fraction (α) are the anatomical features and do not depend on the drug being ingested. We found average values for the normal brain, TS = 1.6 and α = 0.2 (Cserr et al., 1991; Lehmenkühler et al., 1993; Perez-Pinzon et al., 1995; Kume-Kick et al., 2002; Zhu et al., 2020). We calculated the diffusion coefficient for the psilocybin in water at normal brain temperature (37°C) using the well-known linear analytic relationship between molecular weight and diffusion coefficient (Evans et al., 2013, 2018). The diffusion coefficient in the extracellular space (ECS) is 40 percent of the value in the water (Patlak and Fenstermacher, 1975; Fenstermacher and Davson, 1982), therefore, the calculated diffusion coefficient of the psilocybin in the ECS is 3.052 × 10^–10^ m/s.

We calculated (i) brain-to-blood transfer coefficient (K_*pi*_) and (ii) blood-to-brain transfer coefficient (K_*po*_) for psilocybin, based on experimental observations from other drugs (in the literature, there is an absence of any experimental study which directly measures transfer coefficients for psilocybin). We utilized the brain-to-blood and blood-to-brain transfer coefficient of the Meglumine Iothalamate (molecular weight: 809) (Groothuis et al., 1990), Iopamidol (MW:777) (Ivan Yeung et al., 1992) and alpha-aminoisobutyric acid (molecular weight: 104) (Blasberg et al., 1983), to calculate K_*pi*_ and K_*po*_ of the psilocybin (molecular weight: 284.25) using the linear interpolation technique. After mathematical analysis, we obtained K_*pi*_ = 16.38 × 10^–3^ per min and K_*po*_ = 1.4 × 10^–3^ ml/g of tissue/min for psilocybin.

Next, we found out the average psilocybin concentration in blood (C_*a*_) by assessing from seven healthy subjects in a different study recently, where the assay was performed using high-performance liquid chromatography after oral ingestion of the psilocybin (Lindenblatt et al., 1998). Thereafter, we applied the finite difference method for the partial differential equation to solve Equation (9) with a temporal step size of 0.01 min, a spatial step size (three dimensions) of 0.01 centimeters. We approximated the spatial volume as equal to the average brain volume (∼1300 cm^3^) (Ricard et al., 2010). We wrote a python program and calculated the temporal variation of the psilocybin concentration in the brain for 2000 min, i.e., about 33 h, which is comparatively a long duration, and by that time vast majority of psilocybin had been excreted by the subject (The corresponding codes are available at https://github.com/pratik-purohit/modulation-visual-perception). For computing, we utilized the facility of the National Supercomputing Mission of the Govt. of India (Institutional Param-Shivay Supercomputer facility).

#### 3.1.2. Chlorpromazine

We utilized different parameters regarding the chlorpromazine dynamics in the brain from various experimental studies, to solve Equation (9). For instance, the average values of tortuosity (TS) and volume fraction (α) for the normal brain are 1.6 and 0.2, respectively. Then, we calculated the diffusion coefficient for chlorpromazine in water at 37°C using the same method used for psilocybin in section “3.1.1. Psilocybin” and arrived at our derivation of D (chlorpromazine in ECS) = 2.9 × 10^–10^ m/s. Furthermore, we derived k_*pi*_ = 16.65 × 10^–3^ ml/g of tissue/min and k_*po*_ = 1.43 × 10^–3^ per min for chlorpromazine using the method explained in section “3.1.1. Psilocybin.” Next, we availed of the average chlorpromazine concentration in the blood through information from experimental measurements from the eleven healthy subjects using the extraction radioimmunoassay method after oral ingestion of the 100 mg chlorpromazine (Yeung et al., 1993). Thence, we applied the finite difference method for the partial differential equation to solve Equation (9) with a temporal step size of 0.01 min, spatial step size (three dimensions) of 0.01 centimeters, and approximated the spatial volume equal to the average brain volume (∼1300 cm^3^) (Ricard et al., 2010). We constructed a python program and calculated the temporal variation of the chlorpromazine concentration in the brain for 2500 min. i.e., about 41 h, which is a substantial duration, and by that time virtually vast majority of chlorpromazine has been eliminated by the subject (The corresponding codes are available at: https://github.com/pratik-purohit/modulation-visual-perception).

### 3.2. Diffusion MRI tractography

The diffusion weighted MRI scans of 30 subjects (cognitive normal) were randomly selected from the Open Access Series of Imaging Studies (OASIS) image bank (LaMontagne et al., 2019). Ethics committee approval was given by the Knight Alzheimer’s Disease Research Center at Washington University, St. Louis, MO, USA. The diffusion MRI scans were rotated to align with the AC-PC line. The accuracy of b-table orientation was examined by comparing fiber orientations with those of a population-averaged template (Yeh et al., 2018). The tensor metrics were calculated using DWI with *b*-value lower than 1750 s/mm^2^. A deterministic fiber tracking algorithm (Yeh et al., 2013) was used. After preprocessing, we used the following tracking parameters in DSI Studio software (https://dsi-studio.labsolver.org/) to find the neural tracts: anisotropy threshold was 0.04162, the angular threshold was 70 degrees, the step size was 0.01 mm, and a total of 100,000 seeds were placed.

### 3.3. Neuronal network model of grid cells

Let us consider a neural network with continuous attractor dynamics that participate in mapping the visual space with connections between nodes in the neural network to form a toroidal topology. Based on the experimental observations (Killian et al., 2012; Nau et al., 2018), we can take that eyeball movement (with head-fixed) provide information about the relative position in the external visual field to the neural network {similar to the self-motion information [path integration (Etienne and Jeffery, 2004)] in the case of the navigation space (McNaughton et al., 2006)}. Now we are constructing a neuronal network model inspired by the computational models of grid cells (McNaughton et al., 2006; Guanella et al., 2007; Burak and Fiete, 2009). The activity of a node P_*j*_ at time t+Δt is as follows [Equation (10)], where N is the total number of nodes and λ is stabilization strength, where the nodes of the neural network forming a torus topology (Guanella et al., 2007).


(10) $$P_{j}\,\left(t+△\,t\right)=\left(1-\lambda \right)\,\sum_{i=1}^{i=N}P_{i}\,\left(t\right)\,W_{i\,j}\,\left(t\right)$$

$$+\lambda \,\left(\frac{\sum_{i=1}^{i=N}P_{i}\,\left(t\right)\,W_{i\,j}\,\left(t\right)}{\sum_{i=1}^{i=N}P_{i}\,\left(t\right)}\right)$$

Equation (10) shows that the W_*ij*_ is the connection weight between node P_*i*_ and P_*j*_. As shown by Equation (11) below, the weight (W_*ij*_) follows the gaussian function, which depends on the toroidal distance (∥ … ∥_*toroidal*_) between the node P_*i*_ and P_*j*_ (McNaughton et al., 2006), and eyeball speed vector (E(t) = E_θ_, E_Φ_ where θ and Φ are angular coordinates of the fixation point). T determines the ratio of the excitatory and inhibitory connections, and I affects the interaction strength between the node P_*i*_ and P_*j*_ while Z_*i*_ and Z_*j*_ are the coordinates of the node P_*i*_ and P_*j*_ on the torus. The equations mentioned above [Equations (10) and (11)] are reformatted from Equations 1, 2, and 14 of Guanella et al. (2007), specific to our case.


(11) $$W_{i\,j}\,\left(t\right)=I\,e^{-\left(\frac{{∥z_{i}-z_{j}+E\,\left(t\right)∥}_{t\,o\,r\,o\,i\,d\,a\,l}^{2}}{s^{2}}\right)}-T$$

We simulated a neural network-based model of the grid cells, where nodes (grid cells) were arranged on the two-dimensional surface. The total number of nodes were 90 (= 9 × 10). The nodes were arranged on the surface of the torus, as illustrated in Supplementary Figure S4, and the distances between nodes were calculated, accordingly. Therefore, the arrangement of the nodes constituted the periodic boundary. The circumference of the minor and major circles of the torus were 0.5 and 0.61, respectively. Here, one should note that torus topology is defined to assign the weights and connectivity among the nodes (depending on their distances on the toroidal surface). However, the physical positions of the grid cells in the brain may not constitute toroidal topology.

Then, we observed the activity of the above-mentioned neural network model of the grid cell network for the following two cases:

#### 3.3.1. Case-1: normal condition (without any autonomic nervous system activation)

##### 3.3.1.1. Eyes fixating on a target in the visual field

In this condition, the eyeball speed vector is zero [E(t) = 0], and thus W_*ij*_ in this situation was as follows [Equation (12)]. In this case, the weights (W) were constant with time.


(12) $$W_{i\,j}\,\left(t\right)=I\,e^{-\left(\frac{{∥z_{i}-z_{j}∥}_{t\,o\,r\,o\,i\,d\,a\,l}^{2}}{s^{2}}\right)}-T$$

##### 3.3.1.2. Eyeball moving to scan the visual field

In this condition, the eyeballs move with time; therefore, the eyeball speed vector was non-zero [E(t) ≠ 0]. The weights (W) were changing with time [Equation (11)].

#### 3.3.2. Case-2: under drug-induced activation

After the drug ingestion, the drug molecules can modulate the population activity of the grid cell network by binding with the receptors on the grid cell or indirectly through another pathway, as discussed in section “2.1.2. Modulation index.” There transpires to be experimental corroboration of drug-induced alteration of spatial mapping due to modulation of neuronal coding. For instance, experimental electrophysiological studies show that due to the modulatory effect of the excitatory-inhibitory interactions, the synaptic weights of the grid cell network could change (Shipston-Sharman et al., 2016). Another experimental corroboration comes from an empirical investigation (Bonnevie et al., 2013) which demonstrates that there are alterations in the synaptic weights due to the change in grid cell network activity induced by pharmacological agents that produce synaptic modulation, such as muscimol (a GABA agonist and hypoactivation-inducing agent).

Therefore, we can assume that the drug molecules modulate the weight between the nodes of the neural network. However, the modulation of the weights will not be uniform across neural network because the concentration of drug molecules will not be the same at every spatial coordinate in the brain, as indicated by Equation (9). Instead, the synaptic weight modulation shall depend on the local concentration of the drug molecules around the grid cell (node). Hence, to quantify the modulatory effect of the drug molecules, the physical location of the neurons and the drug concentration at that location will be require.

In our model, drug action modulates the weights (W) by affecting the interaction (I) between the nodes. We can express the change in the interaction as node *i* act as input to the node *j* (*$△\,I_{i\,j}$*) as a function (f) of the drug concentration (C) in the infinitesimal volume around node *i.*

Therefore, in mathematical terms, we can write that:


(13) $$W_{i\,j}\,\left(t\right)=\left(I+△\,I_{i\,j}\,\left(t\right)\right)\,e^{-\left(\frac{{∥z_{i}-z_{j}+E\,\left(t\right)∥}_{t\,o\,r\,o\,i\,d\,a\,l}^{2}}{s^{2}}\right)}-T$$

where*:*


(14) $$△\,I_{i\,j}\,\left(t\right)=b.f\,\left(\frac{\int_{x_{i}}^{x_{i}+△\,x}\int_{y_{i}}^{y_{i}+△\,y}\int_{z_{i}}^{z_{i}+△\,z}C\,\left(x,y,z,t\right)\,dx\,dy\,dz}{△\,x\,△\,y\,△\,z}\right)$$

In Equation (14), △x△y△z is an infinitesimal volume around the node (grid cell), (x_*i*_, y_*i*_, z_*i*_) are the physical coordinates of the node P_*i*_ in the brain, and b is a scaling factor. Equation (14) also highlights that the “△*I*_*ij*_″ is a function of the spatial coordinates (x, y, z) and time (t). Further, considering that the neural network is located within a microvolume where the drug distribution can be considered spatially uniform, then “*△Ii⁢j′′* will depend mainly on time (t).

Then, we analyzed the above-mentioned neural network model of the grid cell network under the following conditions.

##### 3.3.2.1. Eyes fixating on a target in the visual field

In this condition, the eyeball speed vector is zero [E(t) = 0], and W_*ij*_ in this situation was as follows [see Equation (11)]. As shown by Equation (15), the weights (W) were changing with time, depending on the drug dynamics.


(15) $$W_{i\,j}\,\left(t\right)=\left(I+△\,I_{i\,j}\,\left(t\right)\right)\,e^{-\left(\frac{{∥z_{i}-z_{j}∥}_{t\,o\,r\,o\,i\,d\,a\,l}^{2}}{s^{2}}\right)}-T$$

##### 3.3.2.2. Eyeball moving to scan the visual field

In this condition, the eyeballs move with time; therefore, the eyeball speed vector was non-zero [E(t)≠0]. The weights (W) were changing with time [Eqs. (13, 14)] due to eyeball movements and the modulatory action of the drug molecules.

Considering the above two cases, we used Equation (10) to calculate the activity of nodes at time t+Δt, which depends on the activity of the network at time t and weights (W), using Δ*t* = 0.01 s and λ = 0.8. The initial activity of the nodes at time *t* = 0 was randomly distributed between 0 and $1/\sqrt{90}$ (total number of nodes = 90). Equation (13) provides the weights between the nodes under the: (i) drug-induced neural activation (ΔI≠0) and (ii) normal condition (no-drug condition, Δ*I* = 0), keeping *s* = 0.24 and *T* = 0.05.

Since there are only 90 grid cells (nodes) in our network, we assumed that the local drug concentration at each node would be almost equal at any instant. Therefore, the weights (W) will be affected equally as the drug concentration changes with time. We evaluated the effect of the drug molecules on the grid cell activity by calculating the network activity for 4 s (time step: 0.01 s) for different values of the ΔI (0 to 0.30). We used python programming language to implement the neural network model of the grid cell (The corresponding codes are available at: https://github.com/pratik-purohit/modulation-visual-perception).

## 4. Results

### 4.1. Validation based on independent experiments

#### 4.1.1. Visual space under psilocybin-induced hyperactivation

**FIGURE 3:**
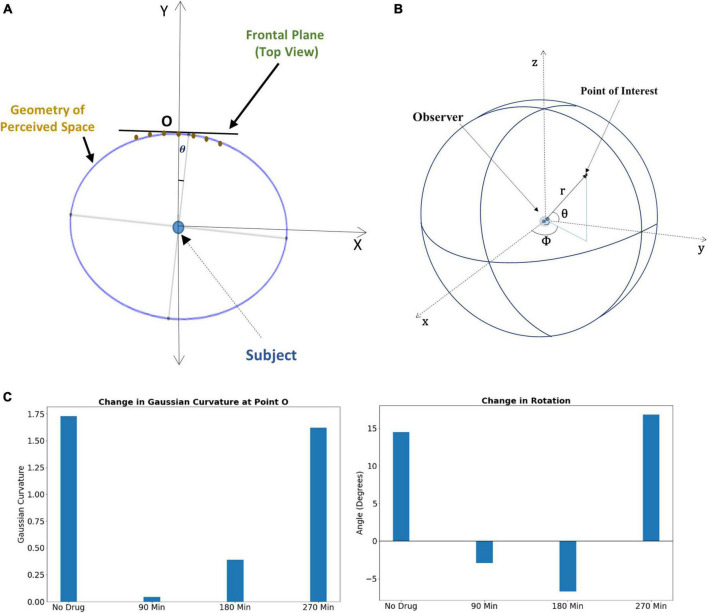
**(A)** Rotated ellipse represents the geometry of the perceived space under psilocybin administration. **(B)** The spherical coordinate system for locating a point in three-dimensional visual space. **(C)** Left panel: Alteration of Gaussian curvature of the perceptual space, the curvature being estimated at the fixation point O. Right panel: Alteration of the rotation angle of the perceptual space, the rotation being estimated from the perspective of the subject at the center of the ellipse. For both panels, the change in the alteration is shown at different time points after psilocybin ingestion.

Subjects under the influence of the psilocybin moved vertical rods to perceive them to be on a flat plane (Supplementary Figure S5); however, they physically positioned them in a substantially curved plane. Under increasing drug influence, the curvature of the curved plane increases further because of the variation in the Psilocybin concentration in the brain. Supplementary section S2 may kindly be referred to, where we have mentioned the methodology of the experiment on the transformation of the apparent frontal plane under the effect of psilocybin. We used these data points for curve fitting and thereby formulated the optimal curve, which follows the physical position of the rods in which the psilocybin action ensues. We find this curve to be a rotated ellipse (Figure 3A), whose rotation angle and Gaussian curvature vary with time, as shown in Figure 3C.

Using the following procedure, we then derived the metric tensor for the surface of the ellipsoid using the parametric equation [Equations (16a), (16b), and (16c)] and the transformation of the metric tensor components to the spherical coordinate system [Equation (11)].

The parametric equations for the ellipsoid are:


(16a) x=a⁢c⁢o⁢s⁢α⁢c⁢o⁢s⁢β

(16b) y=b⁢c⁢o⁢s⁢α⁢s⁢i⁢n⁢β

(16c) z=a⁢s⁢i⁢n⁢α

Tensor transformation equation:


(17) $$g_{\alpha \,\beta }=\sum_{k=1}^{k=3}\sum_{l=1}^{l=3}G_{l\,k}\,\frac{d\,x^{k}}{d\,w^{\alpha }}\,\frac{d\,x^{l}}{d\,w^{\beta }}$$

In Equation (17), G_*lk*_ are the components of metric tensors in the cartesian coordinate system, *x*^i^*: {x, y, z}* coordinates of the Cartesian coordinate system, and *w*^j^*:{α, β}* coordinate of the point on the ellipsoid (similar to Figure 3B). The resultant metric tensor on the surface of the ellipsoid after solving Equation (17) is shown in Equation (18) below [we used Wolfram Mathematica (Version 11, https://www.wolfram.com/mathematica), for computing the metric tensor]:


(18) $$g_{\alpha \,\beta }=\left(\begin{array}{c} E_{1}\,E_{2}\\ E_{3}\,E_{4} \end{array}\right)$$

Here:

*E*_1_ = *a*^2^*cos*^2^*α* + *a*^2^*cos*^2^*βsin*^2^*α* + *b*^2^*sin*^2^*αsin*^2^*β*

*E*_2_ = (*a*^2^ − *b*^2^) *cos α cos β sin α sin β*

*E*_3_ = (*a*^2^ − *b*^2^) *cos α cos β sin α sin β*

*E*_4_ = *cos*^2^ *α*(*b*^2^*cos*^2^*β* + *a*^2^ *sin*^2^ *β*)

where:

*α*, *β*: Angular coordinates of the point on the ellipse.

*a*, *b*: Length of the semi-major and semi-minor axis.

**FIGURE 4:**
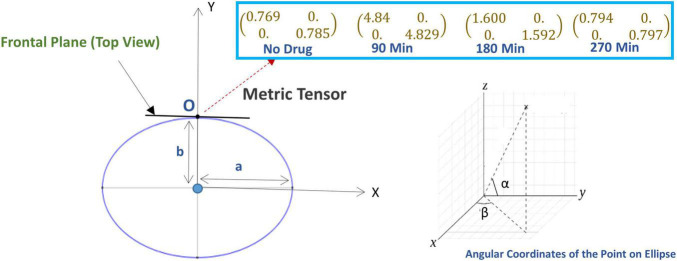
**(Lower left panel)** The ellipse represents the geometry of the perceived space in the horizontal plane where “a” and “b” are the length of the major axis and minor axis (the ellipse is in the horizontal plane). Here, the subject is sitting at the center of the ellipse and looking along the *y*-axis, i.e., looking directly forward, while sitting in the experimental chair with a chin-rest. Before the experiment starts, the vertical rods are arranged in the vertical fronto-parallel plane. **(Lower right panel)** The coordinate system for measuring the angular coordinate of a point on the ellipse. [**Upper right panel** (Inset box)] The alteration of the metric tensor at point O (fixation point), at four successive time points: time *t* = 0 (just before the experiment, no psilocybin given), then at time points *t* = 90, 180, and 270 min after psilocybin ingestion.

We applied Equation (18) and calculated the change in metric tensor at the subject’s visual fixation point O, our results are shown in Figure 4. The metric tensor components vary with the time under the psilocybin influence due to variations in the rotation angle, length of the semi-minor axis, and semi-major axis.

#### 4.1.2. Relationship of perceptual spatial alteration to drug concentration

**FIGURE 5:**
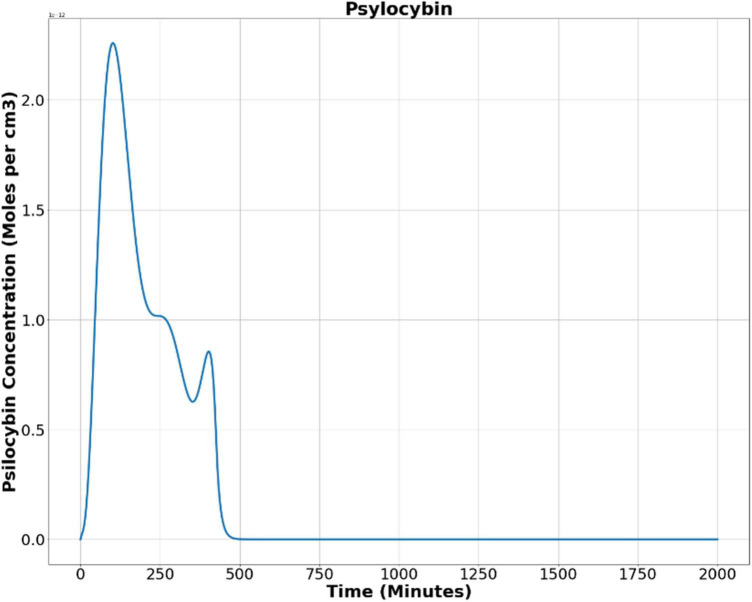
Psilocybin concentration in the brain’s extracellular space after oral ingestion.

Now, we used Equation (9) to find out the psilocybin concentration in the brain. Figure 5 shows the results obtained after solving Equation (9), showing the alteration of psilocybin concentration in brain tissue as time elapses.

**FIGURE 6:**
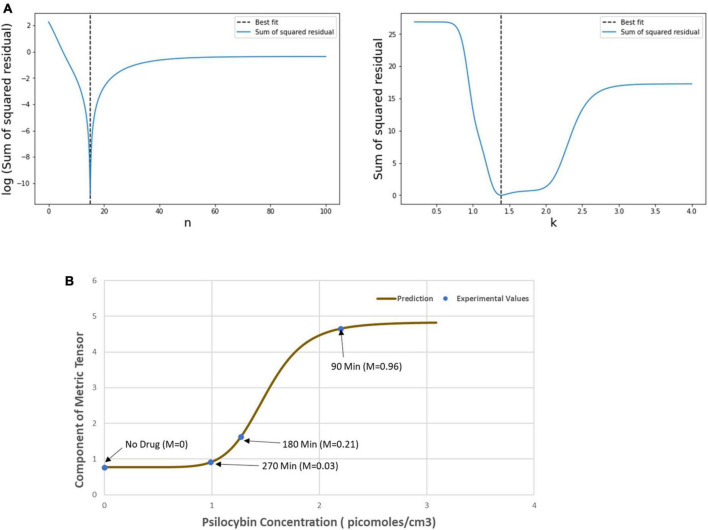
**(A)** Variation of the sum of squared residual with the Hill coefficient (*n*) and the Half-effect drug concentration (*k*). At *n* = 14.8 units (left panel) and *k* = 1.39 picomoles/cm^3^ (right panel), the sum of squared residual is minimum, showing the optimum fit. **(B)** The curve shows the mathematically calculated alterations in the metric tensor component of the perceptual space (solid line) while the psilocybin concentration changes. The filled circles show the experimentally derived metric tensor components along with the corresponding value of the modulation index (*M*) and the time duration since the start of psilocybin ingestion (in minutes). Note the close correspondence of the experimental data-points to the theoretical curve. Indeed, the mathematical model is well validated by the experimentally measured observations, which is further corroborated by robust satisfaction of the goodness-of-fit criterion (χ^2^ statistical test firmly satisfied, *p* > 0.99).

Then, we used the least square method to find the optimum values of the Hill coefficient (n) and half-effective drug concentration (k), which would be able to satisfactorily predict the metric tensor components using Eqs. (5, 6). This procedure we perform is as follows. First, we calculated the sum of square residuals using (i) the mathematically calculated metric tensor components, using Eqs. (5, 6), and (ii) the experimentally derived metric tensor components (Figure 4). Then we plotted the Hill coefficient (n) and half-effect drug concentration (k) against the sum of square residuals, as shown in Figure 6A. As displayed in the two panels of Figure 6A, for *n* = 14.8 units and *k* = 1.39 picomoles/cm^3^, the error between mathematically calculated metric tensor and the experimentally derived metric tensors is minimum, and these two values are thus the optimum values of n and k. We applied this procedure to two diagonal components of the metric tensor and obtained the same results.

After calculating the optimum values of the n and k, we plotted the mathematically calculated value of the metric tensor (the value of both diagonal components were equal) against the experimentally inferred value of the metric tensor (Figure 6B). As shown in Figure 6B, the experimental data-points closely coincide with the theoretically predicted curve, this coincidence we assessed by the goodness-of-fit statistical criterion (χ2-squared test), which showed that the congruence was very robust (*p* > 0.99). This strong congruence thus well validates our computational framework that the temporal alteration of the metric tensor due to psilocybin action follows the temporal trajectory of the modulation index [Equation (6)]. In the next section “4.1.3. Prediction of metric tensor alteration under psilocybin induced hyperactivation,” we will use these values of n and k to predict the outcomes of a different experiment.

#### 4.1.3. Prediction of metric tensor alteration under psilocybin induced hyperactivation

**FIGURE 7:**
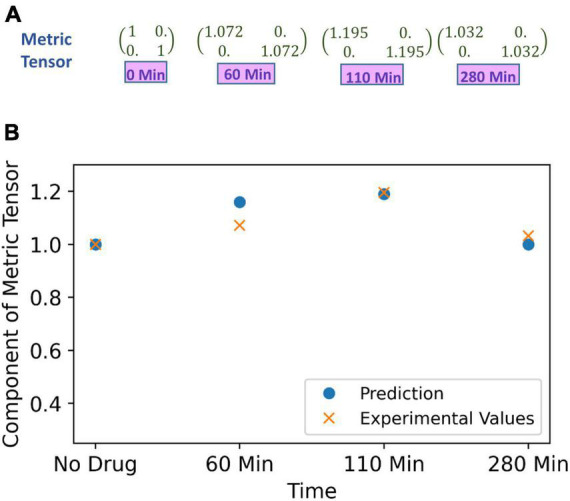
**(A)** Metric tensor of the perceived space obtained from corresponding experimentally measured spatial distortion threshold at four successive time points: time *t* = 0 (just before experiment, no psilocybin given), then at time points *t* = 60, 110 and 280 min after psilocybin ingestion. **(B)** The metric tensor of perceptual space under the psilocybin induced hyperactivation condition. The theoretically computationally predicted values are shown in blue filled circle, while the experimentally derived values are shown by the red cross. Observe the strong congruence of the experimental points with the theoretical points. Actually, the theoretical model is soundly validated by the empirical data as substantiated by strong satisfaction of goodness-of-fit criterion (there is robust satisfaction of the χ^2^-squared statistical test).

To recapitulate, one may kindly give a perusal on Supplementary section S3, where we have mentioned the methodology of the experiment on spatial distortion threshold assessed by an optical prism set-up, when the subject is under effect of psilocybin. The prism lens shifts the object’s apparent position, and changing the prism power with time introduces visual distortion. However, the brain can counter adapt, and the subject does not perceive any distortion. In that experiment, the counter adaption limit was measured by finding the minimum prism power required to perceive distortions under the psilocybin-induced hyperactivation of the nervous system. Hyperactivation reduces the minimum prism power (β) required to perceive deviation from the straightness of the horizontal black line (Supplementary Figure S6). Variation in the hyperactivation level during the experiment causes different values of β to produce the same perception, which denotes their equivalence in the perceptual space. We find out the physical area covered by the black line for different values of the β. Since the black line covers the same area in the perceptual space, we determine the required change in the metric tensor for the equal area. At the beginning of the experiment, without drug ingestion (at 0 min), we can take that the diagonal components of metric tensor are unity, and then we calculated the metric tensor at time 60, 110, and 280 min as follows, the values of the metric tensor that we derived are shown in Figure 7A.

In order to predict the metric tensor components using Eqs. (5, 6), we obtained the psilocybin concentration (C) in the brain at 0, 60, 110, and 280 min using Equation (9). We used the procedure mentioned in the section “3.1.1 Psilocybin” to solve Equation (9). In previous section “4.1.2. Relationship of perceptual spatial alteration to drug concentration.” we calculated the values of n and k for the psilocybin. Since now we have psilocybin concentration (C), then by using Equation (6) and values of *n* = 14.8 and *k* = 1.39 obtained in the previous section, we are able to obtain values of the modulation index M at 0, 60, 110, and 280 min. Thereafter, we derived the components of the metric tensor after applying Equation (5) and the modulation index (M) values. A comparison between the metric tensor component obtained from our experimental calculations and from our theoretical model prediction is shown in Figure 7B, and the χ2 (chi-square) goodness of fit test (*p* > 0.99) verifies that the experimental data well validates the theoretically predicted model. Diagonal components of the metric tensor are equal; therefore, only one component is shown in Figure 7B. Hence, our model can satisfactorily predict the metric tensor components.

#### 4.1.4. Prediction of metric tensor under chlorpromazine induced hypo-activation

**FIGURE 8:**
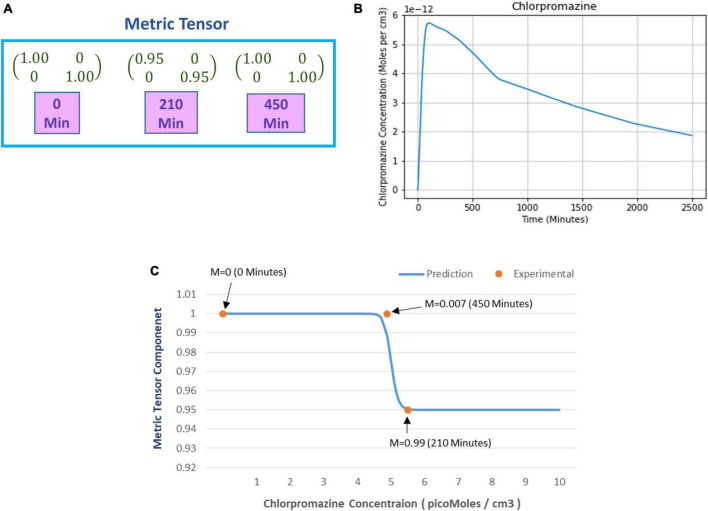
**(A)** Numerical values of the metric tensor components of the perceived space obtained from experimentally recorded spatial distortion threshold at time *t* = 0, 210, and 450 min after ingestion of 50 mg oral intake of chlorpromazine. **(B)** Predicted chlorpromazine concentration (picomoles/cm^3^) in the brain’s extracellular fluid as calculated from the theoretical computational model (chlorpromazine input is 50 mg). **(C)** The curve shows the predicted alteration in the mathematically calculated metric tensor component of the perceptual space (solid line) while the chlorpromazine concentration changes. The filled circles show the experimentally derived data-points estimating the metric tensor components. For each experimental data-point, there is shown the time duration since the start of chlorpromazine ingestion (in minutes). Note the close correspondence of the experimental data-points to the theoretical computational curve. Indeed the mathematical model is well corroborated by the experimentally measured observations.

A similar experimental approach was adopted to find the minimum prism power to perceive distortions but after ingestion of the hypoactivation inducing drug chlorpromazine. The details are provided in Supplementary section S4 which may kindly be referred for recollection. As opposed to hyperactivation, the state of hypoactivation increases the minimum prism power (β) to perceive distortion. We used the same analysis technique (explained in section “4.1.3. Prediction of metric tensor alteration under psilocybin induced hyperactivation”) to compute the metric tensor components for the corresponding prism power, the components of our calculated metric tensor is shown in Figure 8A.

After calculating the metric tensor, we used Equation (9) to determine the chlorpromazine concentration in the brain. Figure 8B shows the results obtained after solving Equation (9) for the chlorpromazine. Now, we use a similar procedure like that we used in section “4.1.2. Relationship of perceptual spatial alteration to drug concentration” for psilocybin. Hence, for chlorpromazine, we used the least-square method to find the optimum values of the Hill coefficient (n) and half-effect drug concentration (k) for the minimization of the difference between (i) mathematically calculated metric tensor components [using Eqs. (5, 14)], and (ii) experimentally derived metric tensor components. We calculated the optimum values of the coefficients n and k. Here, for chlorpromazine, we obtained the *n* = 50.3 units and *k* = 4.96 picomoles/cc^3^. One may observe that n and k are different in chlorpromazine and psilocybin (see section “4.1.3. Prediction of metric tensor alteration under psilocybin induced hyperactivation”), this reflects the underlying pharmacokinetic and drug potency differences. In Figure 8C, using the n and k values of chlorpromazine, we plotted (i) the experimental values (dots) of the metric tesor, and (ii) the mathematically predicted value of the metric tensor (continuous line).

From Figure 8C we observe that the experimentally determined metric tensor components well corroborate the theoretically predicted metric tensor components, as chlorpromazine concentration increases. The close congruence between the experimental data points and the theoretical computational curve in Figure 8C may be observed. [In parenthesis, it may be mentioned that there are four data points for the psilocybin experiment in Figure 6B, whereas there are three data-points for the chlorpromazine experiment in Figure 8C. It might have been suitable if the chlorpromazine study had also four data points, nevertheless in this experiment with hypoarousal-inducing chlorpromazine, the study logistics afforded the measurement of only three temporal data points on the individual subjects]. Note that Figure 8C shows a sigmoid-type decreasing curve for chlorpromazine, and the pattern is reciprocal to Figure 6B that displays a sigmoid-type increasing curve for psilocybin. This reciprocity is a manifestation of the neurophysiological complementarity between chlorpromazine (hypoactivation agent) and psilocybin (hyperactivation agent).

### 4.2. Anatomical connectivity between the entorhinal cortex and visual cortex (area V2)

**FIGURE 9:**
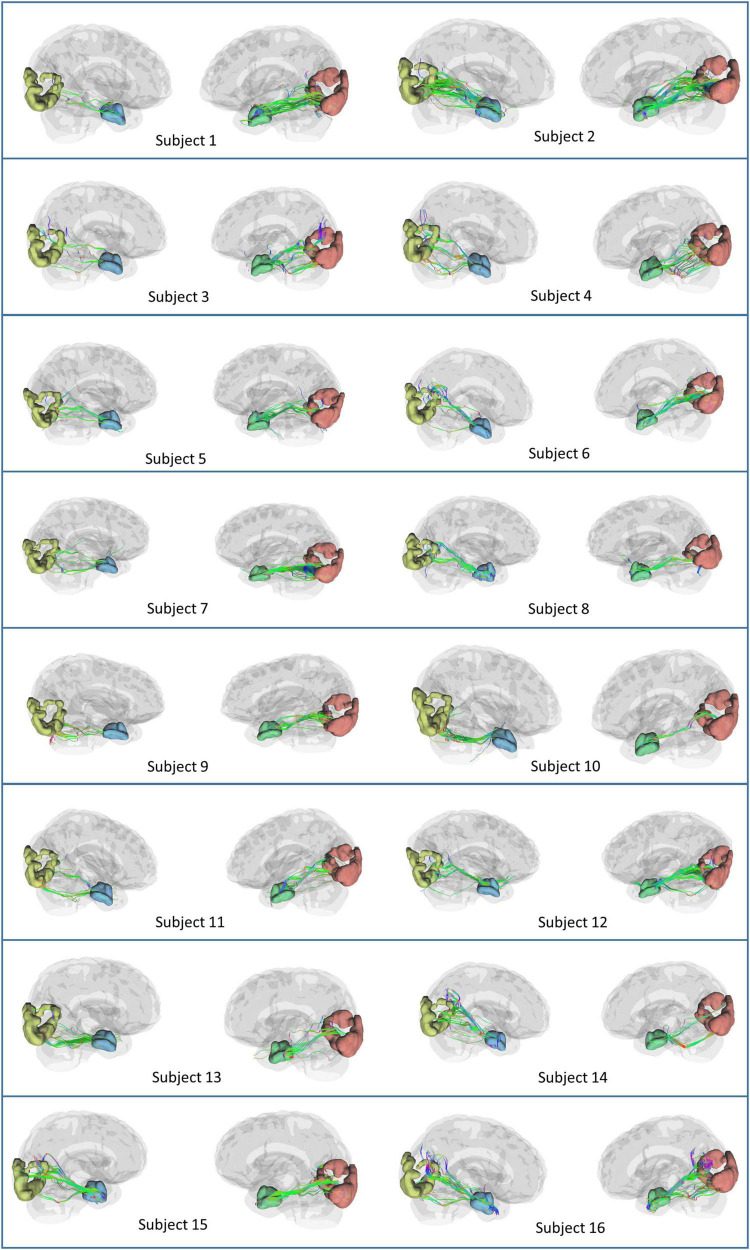
Anatomical connectivity (neural tracts) between the entorhinal cortex and visual cortex (area V2) in the sixteen subjects obtained after performing the tractography experiment. The neural tracts in the remaining fourteen subjects are shown in Supplementary Figure S7.

Based on the theoretical and experimental studies, we have sketched a framework for a grid cell-like encoding of the perception related to various sensory modalities (Supplementary Figure S3). Indeed, grid cells are experimentally recorded from area V2 (Long et al., 2021). Since the current study focuses on visual-spatial perception, we performed an MRI tractography experiment to find the anatomical connectivity between the entorhinal cortex and visual area V2 to verify our theoretical formulations (section 2.2). Using the methodology explained in section “3.2. Diffusion MRI tractography,” we conducted diffusion MRI scan-based tractography for thirty normal subjects. The tracts obtained after analyzing the diffusion MRI scan are visualized in Figure 9 for 16 subjects, and tracts of the remaining 14 subjects are shown in the Supplementary Figure S7. Results distinctly depicted the presence of the neuronal pathways between the entorhinal cortex and area V2. The quantitative parameters related to the neural tracts are shown in Figure 9 are mentioned in Table 1 for each hemisphere of the thirty subjects. The tractography results provide evidence of the anatomical connectivity between the entorhinal cortex and visual cortex (area V2), thus supporting our theoretical formulation. We have also analyzed a 7 Tesla diffusion MRI scan to check the in-principle conformity of our result under a different field strength of the MRI machine. The details and results of the 7-tesla tractography experiment are shown in Supplementary section S5 and Supplementary Figure S8. We obtained similar results in the case of a 7-tesla MRI as well.

**TABLE 1:** Different parameters related to the neural tracts obtained between the entorhinal cortex and area V2.

Parameters	Left hemisphere	Right hemisphere
Mean length (mm)	116.8 ± 4.43	118.5 ± 5.42
Fractional anisotropy	0.2921 ± 0.04	0.2784 ± 0.03
Mean diffusivity	1.086 ± 0.12	1.1531 ± 0.15
Axial diffusivity	1.4013 ± 0.13	1.4572 ± 0.17
Radial diffusivity	0.9375 ± 0.13	1.009 ± 0.15

### 4.3. Neuronal network basis of drug-induced modulation of spatial perception

We have theorized the role of the grid cell-like representation of visual spatial perception in section “2.2. Neural correlates of drug-induced modulation of visual-spatial perception” based on various experimental and computational studies. Here, we constructed a neuronal network model of the grid cells (see section “3.3. Neuronal network model of grid cells” for details) and simulated the modulatory effect of the drug molecules. We calculated the grid cell activity alterations under normal (no drug) and drug-induced activation conditions; the results are presented in the following subsections “4.3.1. During normal conditions (no drug condition)” and “4.3.2. Under drug-induced neural activation.”

#### 4.3.1. During normal conditions (no drug condition)

**FIGURE 10:**
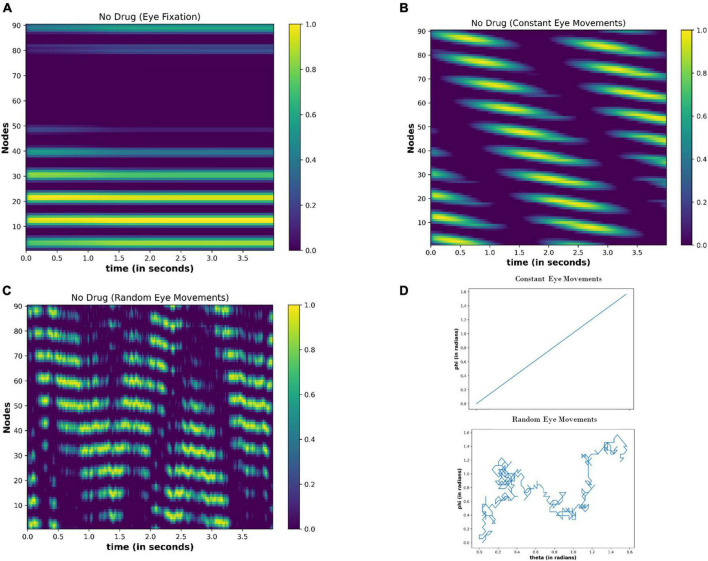
Activity of the neuronal nodes under no-drug condition **(A)** when eyes are not moving and fixating in the visual field **(B)** when eyes are making a constant movement in the visual field **(C)** when eyes are randomly scanning in the visual field. **(D)** Variation in the angular position of the eyeball movements during constant eye movement (upper) and random eye movement (lower).

Under no drug condition (Δ*I* = 0), we calculated the activity of the nodes for the 4 s (time step = 0.01 s) for the following cases: (i) Eyes fixating in a visual field [E(t) = 0] (ii) Eyeballs constantly moving [E(t) = constant] (iii) Random movement of the eyes [E(t) = random]. Figure 10 shows the results after the simulation. When eyes are not moving (fixating), the few nodes are constantly active independent of the time (Figure 10A), thereby showing the attractor dynamics of the neuronal network. However, the activity of the nodes varies with time (Figures 10B, C) during the eyeball movements. When eyeballs are moving with constant speed, the periodic activation of the nodes is apparent. However, in the case of random movements, the activity of the nodes seems random with time.

#### 4.3.2. Under drug-induced neural activation

We found out the effect of the drug-induced neural activation on the grid cell activity by changing the △I from 0 to 0.30. △*I* = 0 is equivalent to the no drug condition, and non-zero values of the △I represent the drug-induced activation. We simulated the neural network model of the grid cells for 4 s (time step: 0.01). Similar to the no-drug situation, we evaluated the network activity in three cases (eye fixation, constant eye movements, and random eye movements); the results are as follows:

##### 4.3.2.1. Eyeballs fixation (no eye movement)

**FIGURE 11:**
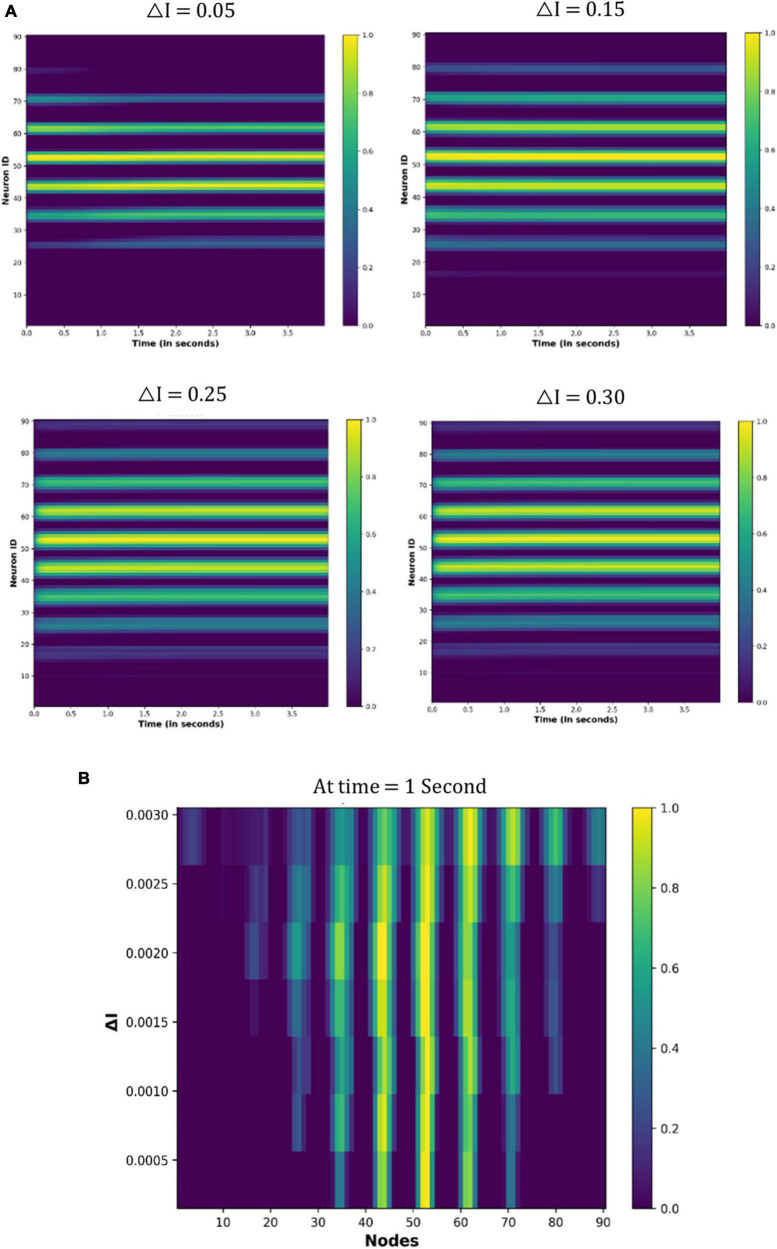
Activity of the neuronal nodes under drug-induced activation: **(A)** activation pattern of the nodes under the different values of the △I = 0.05, 0.15, 0.25, and 0.30. **(B)** Variation in the activity of node number 47 as the △I changes from 0 to 0.30.

Figure 11 shows the node activity under different △I values. Because eyes are not moving, a few nodes are constantly active for particular values of the △I (Figure 11A). However, different sets of nodes are active as the △I changes (Figure 11B). These observations indicate that the neuronal coding of the visual spatial perception is changing as drug-induced neural activation (△I) varies.

##### 4.3.2.2. Constant eye movements

**FIGURE 12:**
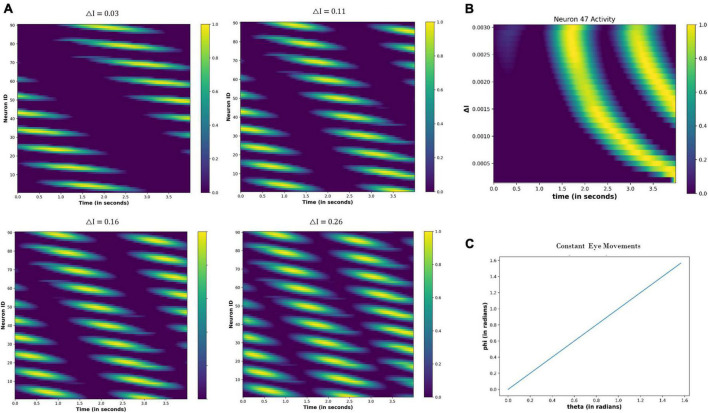
**(A)** Change in the periodic activation of the neuronal nodes for different values of the △I. **(B)** Activity of the neuronal node 47 for the different values of the △I and time. **(C)** Variation in the angular coordinates of the moving fixation point. At the origin, *t* = 0 s: θ = 0 and Φ = 0; thereafter, θ and Φ are increasing linearly with time, indicating that eyes move with a constant velocity across both angular directions.

Figure 12 shows the neural network activity under different values of the △I. The nodes show periodic activation, whose period decreases as the △I increases (Figure 12A). Thus, the neural activation affects the firing pattern of the nodes, as highlighted in Figure 12B, by showing the variation in the activity of node 47 as the △I changes.

##### 4.3.2.3. Random eye movements

**FIGURE 13:**
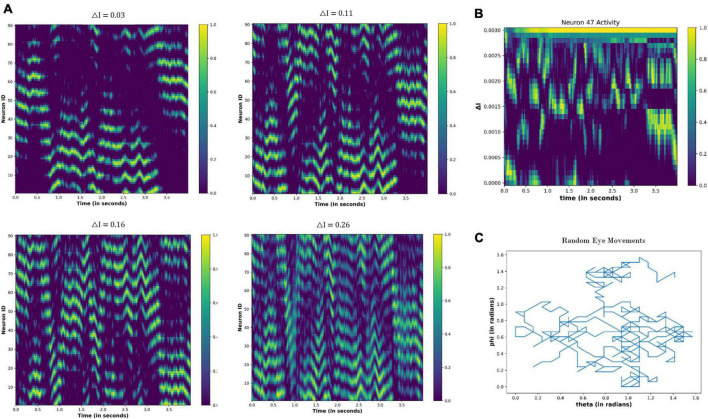
**(A)** Activity pattern of the neuronal nodes for the different values of the △I, as the eyeball is moving randomly in the visual field. **(B)** Temporal variation in the activity of neuronal node 47 for the different values of △I. **(C)** Variation in the angular coordinates of the randomly moving fixation point.

When the eyeballs randomly scan the visual field, the resultant network activity is shown in Figure 13. Contrary to Figure 12, the periodic activity of the nodes is not visually observable. However, as the △I increases, temporal variation in the node activity becomes prominent (Figures 13A, B).

#### 4.3.3. Quantification of the drug-induced neural activation

**FIGURE 14:**
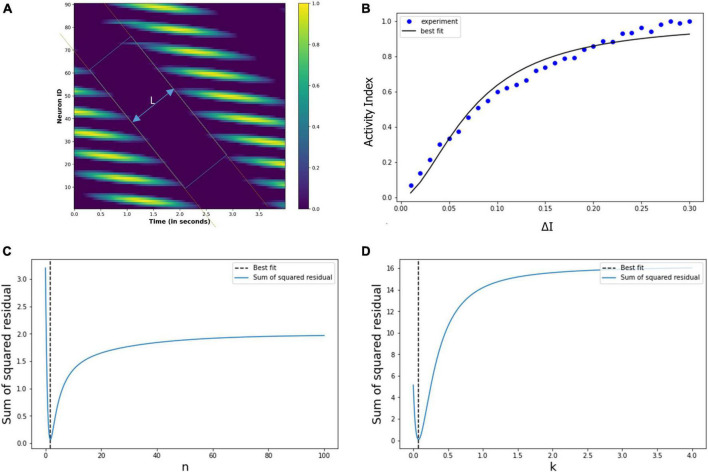
**(A)** Spatiotemporal distance (*L*) for a particular value of the △I. We calculated *L* for the different values of the △I. **(B)** Experimentally calculated activity index (filled circles) using the neural network-based model of grid cell network, and the corresponding Hill equation curve is also shown. **(C,D)** Variation of the sum of squared residual with the Hill coefficient (*n*) and *k*. At *n* = 1.8 and *k* = 0.07, the sum of squared residuals is minimum and optimally fits the observed data points.

In the previous section “4.3.2. Under drug-induced neural activation,” we showed modulation of the grid cell activity due to altered neural activation (△I). In order to quantify the modulatory effect of the drug molecules, we are considering the simple case of constant eye movement during which the nodes showed periodic activity (Figure 12A). Figure 12A shows the spatiotemporal activity of a grid cell network. We measured the distance (L) between the periodic activation of the nodes using MATLAB’s Image Viewer tool (Mathworks Inc.) for the different values of the △I, as shown in Figure 14A [The different values of *L*, as △I varies, are graphically shown in the video which is part of additional files]. After that, we defined an activity index (AI) for quantifying the modulation of the grid-cell network activity, such that the value of the AI ranges between 0 to 1, whereby the AI can be delineated as in Equation (19) below, where L_*max*_ is the maximum value of the L:


(19) $$A\,I=1-\frac{L}{L_{m\,a\,x}}$$

Thence, we calculated the activity index (AI) for each value of the △I, whereby we obtained the data points (filled circles) in Figure 14B. It is evident that the pattern of these points is a saturation function, and so we explored whether these points follow a Hill equation. Then, we used the least square method to fit the variation in the activity index (AI) with the following Hill equation:


(20) $$A\,I=\frac{1}{1+{\left(\frac{k}{△\,I}\right)}^{n}}$$

Thereby, we obtained the optimum values of the Hill coefficient *n* = 1.8 and *k* = 0.07, the curve of this equation is drawn in Figure 14B. We note the close correspondence between the data points and the Hill equation curve (the χ2 test is well satisfied, with *p* > 0.98). Figures 14C, D show the sum of squared residuals for different values of n (Hill coefficient) and **k,** respectively, and elucidates that for *n* = 1.8 and *k* = 0.07, the sum of squared residuals is minimum for which the Hill equation optimally fits the data points (filled circles) of the activity index, AI, in Figure 14B.

Now, we recapitulate the modulation of visual perception under pharmacological action [Equation (6)]. To recollect, the modulation index (M) represents the modulatory effect of drug-induced activation on visual-spatial perception. As shown in Equation (6), the Hill equation there shows the mathematical relationship between the modulation index (M) and the drug concentration (C), which we are re-writing as follows:


(21) $$M=\frac{1}{1+{\left(\frac{k}{C}\right)}^{n}}$$

Let us give attention to the two formulations of the Hill equation, namely (i) Equation (21), which shows how rising drug concentration (C) increases the modulation index (M) that becomes gradually saturated, and (ii) Equation of 20, which shows that as the interaction index (△I) increases, there is a rise in the activity index (AI) which gradually saturates. In other words, Equation (20) can be taken as that when the drug-induced interaction rises, then there is a gradual increase in the neural network activity. Thus, we see the close equivalence between Eqs. (6, 20), both describing the effect of the drug on the neural system from two perspectives: (a) Equation (20) describes the perceptual changes at the neuronal network dynamics level, while (b) Equation (6) describes visuospatial changes at the perceptual-behavioral level (see the last paragraph of section “2.1.2. Modulation index”). In other words, the Hill model can unitarily elucidate visuospatial changes with neuromodulatory chemicals across scales, i.e., neuronal level and behavioral/perceptual level.

## 5. Discussion

### 5.1. Quantitative model

As per the ecological approach to psychological dynamics, the central nervous system furnishes as a perception-action system, whereby the neural system cognizes the surroundings and acts on or adapts to the environment, so as to optimize the integrated functioning of the organism. The sympathetic and parasympathetic nervous system controls the human body’s response to external situation, which is necessary for survival and homeostasis. However, this also manifests in the change in the organization and comprehension of sensory information, termed as perception. We focused on visual perception in this study, although our methodology can be extended to other sensory modalities. Since it is not easy to experimentally quantify and control naturally induced hyperactivation and hypoactivation of the central nervous system (e.g., stress or stupor), we focused on the pharmacologically induced hyper or hypo-activation, which is quantifiable and controllable based on the dosage amount.

We devised a mathematical coefficient: the modulation index (M), whose value can vary between 0 and 1. The modulation index is a single parameter representing the perceptual alterations due to the neuropharmacological action. We elucidated that the activation level (i.e., dose amount) and perceptual alteration (i.e., modulation index) are related to each by the Hill equation [Equation (6)], since perceptual alterations occur due to the biochemical process between drug molecules and receptors. We formalized the metric tensor characterizing the geometrical properties of the visual space, and defined how these metric tensor components will vary with the modulation index (M) [in Equation (5)]. Hyperactivation and hypoactivation conditions cause perceptual deviations in opposite directions, as shown by the arousal index μ being +1 and **−**1, respectively, for hyper and hypo-activational drugs. Further, the intensity of the perceptual distortion depends on the individual’s tolerance to the drug as characterized by the personalized weber constant P [Equation (5) shows the incorporation of both parameters μ and P]. We quantified the level of hyperactivation and hypoactivation by initially formulating a differential equation [Equation (9)] describing the spatiotemporal dynamics of the drug in the brain. Thereafter, we calculated the temporal variation in the metric tensor of the visual space after the oral ingestion of the psilocybin (hyperactivation inducing drug). Thence, we found out the value of the Hill coefficient (n) and half-effective drug concentration (k) [Equation (6)], using experimental observations on psilocybin administered subjects.

### 5.2. Verification by empirical findings

Moreover, drugs, even if they stimulate the same type of activation pathways, may have different bioavailability and biochemical reaction dynamics. Therefore the value of n and k may vary with different drugs. We applied the calculate values of the Hill coefficient (n) and half effective drug concentration (k) from one experiment to another experimental investigation measuring the spatial distortion threshold under psilocybin action. We predicted the metric tensor at different time points using Eqs. (5, 6). Indeed, we found out that the experimental values well validate our prediction. To corroborate this validation, the statistical goodness-of-fit assessment (χ2-test) was performed, and the corroboration was calculated to be really strong. Using a similar methodology, we found the values of the Hill coefficient and half-effective drug concentration for another different experiment, which involved assessing spatial perceptual distortion under the hypoactivation inducing drug, chlorpromazine. The theoretical prediction curve was also closely substantiated by the experimental data points.

### 5.3. Practical perspective

Our various types of experimental validation proved that the variations in the geometry of the visual space due to the modulation of the neural activity (due to the pharmacological agent) could be precisely mathematically modeled using our formulations. In our approach, we used the drug concentration in the brain as an indicator of the neural activation level, the pharmacological activity follows the Hill equation and modulates the perception. We also need to extend the applicability of activation-induced spatial distortion to the practical consequences of altered activation due to environmental or endogenous factors, such as stress, bipolar conditions, stupor, or depressive affect. Therefore, applying our model to the application-oriented scenario would be very useful. For this, there should be a parameter that denotes the activation level (similar to the drug concentration in the brain at any moment), and the parameter should vary with time according to the activation level. A suitable candidate for this parameter is the quantitative variability index in the EEG signal, such as Goldstein’s coefficient of variation (G) across different frequency bands (Fischer, 1971; Goldstein, 1978). In addition to finding a suitable parameter denoting the activation level, the relationship between the drug-induced perceptual modulation index (M) and the activation level parameter G needs to be formalized, derived, and approximated.

### 5.4. Neuron-level processes

We provided a grid cell oriented framework to account for a possible underlying neuronal principle behind the modulation of visual space geometry due to drug-induced autonomic nervous system activation. Our analysis indicates that the local spatial map at the visual cortex and global spatial map at the entorhinal cortex is represented by the grid cells at the neuronal level. As verified and highlighted by our MRI-tractography experiment, the entorhinal-hippocampal network is anatomically linked with the visual cortex (area V2), thereby may be enabling the integration of sensory information with prior knowledge (memory), so as to encode different positions in the visual space. We indicated that nervous system activation alters the interaction between the grid cells in the network, inducing changes in the metric representation of the physical space into the perceived space.

Various computational models of the grid cells have been developed over the years (McNaughton et al., 2006; Guanella et al., 2007; Burak and Fiete, 2009), and based on these models, we constructed and simulated a grid cell network under the drug action. The simulated action of the drug molecules altered the grid-cell firing characteristics, thereby indicating the modification of the neuronal representation of the external visual field, which may manifest as the modulation of the visual-spatial perception. We devised a new parameter (activity index) to quantify the changes in the activity of the grid cell network and found that the activity index (AI) also follows the Hill equation [similar to the modulation index in Equation (6)]. Using the neuronal network model of the grid cell network, we showed that the drug-induced alterations in the visual spatial perception follow the Hill equation as predicted by our mathematical formulation (see section “2. Theoretical analysis and mathematical modeling”).

Moreover, we plan to pursue in-depth collaborative experimental studies to understand detailed dynamics of visual spatial perception at the behavioral and neuronal level under altered neuropharmacological activation levels. As per our knowledge, no experiment has been carried out to record the activity of the grid cells under varying neuropharmacological effects. Indeed, advancement of knowledge about modulation and distortion of visual perception may be investigated in the future such as: (i) fMRI experiment on humans under the influence of drugs that affects the activation level while performing a visual task, and (ii) the experimental recording in monkeys doing visual tasks, under different pharmacologically induced activation levels. Recently, ultrahigh density electrophysiological probes as neuropixel technology (Jun et al., 2017; van Daal et al., 2021; Paulk et al., 2022), may help elucidate some of the aforesaid aspects.

### 5.5. Spectrum of perceptual modulation

Our model provides the computational foundation, validated by the independent experimental observation, of quantifying the perceptual alteration due to the altered neural activation. In other words, our study specifies a quantitative deviation compared to the baseline perceptual levels, which correlates with the cerebral arousal level. Here, we focus on the visual system and show that the metric tensor of the perceived geometry can vary, which manifests as variations in visual assessment, its distortion, and, accordingly, the subject’s gauging of the environment. In principle, we showed that grid cell like neuronal representation map perceived visual space and pharmacological agents affect the grid cell firing characteristics resulting in the modulation of the visual-spatial perception. Additionally, our model has an incisive potential to apply to the most general case of the naturally occurring variations in cerebral arousal, either hyperarousal states as stress, or hypoarousal states as trance. Indeed, we have indicated that our approach can also be applied to arousal-induced distortion of perceptual space of different sensory modalities, such as visual space, auditory space, olfactory space, and sensory-motor space.

A primary rationale for the applicability of our framework to multimodal sensory systems is that grid cells-like neural representations exist while navigating in the visual space, olfactory space, auditory space, and sensorimotor space, this implies a general nature of the grid cells to represent cognitive maps being generated from different types of the perceptual modalities. Our quantitative predictive model to forecast perceptual distortion and altered judgment may provide a novel approach to assessing and improving human performance. In high-stress professions such as aircraft pilots, submarine navigators, and spacecraft astronauts, high arousal can often induce hyperactivation and cause an error of judgment and mishap due to changes in visual perception or auditory perception (Wiegmann et al., 2005; Harris, 2016). By measuring the cerebral arousal level, one can devise an algorithm and alarm to give feedback to the human operator to avoid accidents.

### 5.6. Biomedical implications

Using the reverse approach, experimental measurements of the metric tensor (such as by measuring handwriting alteration) can be used as a novel, rapid, inexpensive paper/pencil behavioral-psychometric test to assess the level of cerebral arousal, thereby detecting and monitoring depressive disorders (hypoactivation) or manic disorders (hyperactivation), or their alternating combination, as a cyclothymic disorder. It is also known (Fischer, 1967) that mood disorders, such as bipolar psychosis, produce, respectively, expansion and contraction of the spatial area of handwriting during the manic phase and the depressive phase. This cited investigation (Fischer, 1967; Nolen et al., 2020) has highlighted that the bipolar disorder of the artist Van Gogh can be well-mapped and analyzed in terms of the geometric alteration in his art and handwriting as time progressed. Indeed, our proposed aforesaid behavioral testing has considerable potential to be used as a ready economical biomarker to monitor the therapeutic effect of clinical interventions on bipolar or mood disorder patients. Such behavioral testing can be utilized on a day-to-day basis for close monitoring and can supplement invasive blood-based biomarkers (García-Gutiérrez et al., 2020) to check the effects of the psychiatric drugs administered; indeed, intravenous blood-based biomarkers may not often be measured on a day-to-day basis.

Further, visuospatial tests to measure the metric tensor components of the perceived space may be designed, and a readily affordable planimetry-based test may be developed to measure the handwriting area at different times. Recent *in silico* models of neuropsychiatric patients and therapeutic intervention optimization are available to assess behavioral therapeutic approaches (Hoffman et al., 2011; Huys et al., 2021). Hence, it may be indicated that our mathematical spatiotemporal analysis could improve the existing available neuroinformatics models of simulated patients for developing more precise quantitative computational models of neuropsychological disorders. As large electrophysiological datasets, genomic maps, and digital therapeutic records become readily available, it may be worthwhile to develop *in silico* models of behavioral, and neuropsychiatric effects and their alteration by clinical interventions.

## 6. Conclusion

The ecological approach to psychological dynamics posits the central nervous system as a perception-action system, with the sympathetic and parasympathetic nervous systems controlling the body’s response to external situations, leading to changes in sensory information processing. This study focuses on visual perception and introduces the modulation index (M) as a mathematical coefficient representing perceptual alterations due to pharmacologically induced hyper or hypo-activation. The relationship between the activation level and perceptual alteration is described by the Hill equation, and the geometrical properties of visual space are characterized by the proposed metric tensor. The personalized Weber constant (P) and arousal index (μ) further affect the intensity of perceptual distortion, with hyperactivation and hypoactivation conditions causing perceptual deviations in opposite directions. The Hill coefficient (n) and half-effective drug concentration (k) can vary with different drugs due to differences in bioavailability and biochemical reaction dynamics. The methodology of the study includes formulating a differential equation to calculate the spatiotemporal dynamics of the drug in the brain and calculating the temporal variation in the metric tensor of visual space. The predicted metric tensor closely matched the experimental data in separate experiments under the effects of psilocybin and chlorpromazine, respectively, with strong statistical validation through the goodness-of-fit test.

Our methodology can be extended to other sensory modalities, and the findings have implications for understanding the effects of neuromodulating agents on perception and cognition. Our analysis suggests that grid cells in the entorhinal-hippocampal network, which has anatomical connectivity with the visual cortex as showed by the tractography experiment, represent the local and global spatial maps at the neuronal level, enabling the integration of sensory information with prior knowledge. By simulating a grid cell network under drug action, we found that drug molecules alter the firing characteristics of the grid cells and modify the neuronal representation of the external visual field, leading to the modulation of visual-spatial perception. The grid cell-like neural representations in different perceptual modalities indicate the general nature of our approach. Our study has developed a computational foundation for quantifying the perceptual alteration caused by altered neural activation, validated by independent experimental observations. The possible practical utility of our study can be: (a) The experimental measurements of the metric tensor, such as by analyzing handwriting alteration, can be used as a novel and inexpensive behavioral-psychometric test to assess cerebral arousal levels and monitor mood disorders like bipolar and depressive disorders. (b) By measuring the cerebral arousal level, one can devise an algorithm and alarm to give feedback to improve human performance in high-stress professions.

## Data availability statement

The original contributions presented in this study are included in the article/Supplementary material, further inquiries can be directed to the corresponding author at Shiv Nadar University (SNU), Greater Noida, India.

## Ethics statement

The studies involving human participants were reviewed and approved by the Knight Alzheimer’s Disease Research Center at Washington University, St. Louis, MO, United States for 3-T MRI subjects and Ethics Committee of the Faculty of Psychology and Neuroscience at Maastricht University, Netherlands (reference number: ERCPN167_09_05_2016) for 7-T MRI subject. The patients/participants provided their written informed consent to participate in this study.

## Author contributions

PP, PD, and PKR: conceptualization, methodology, software, validation, formal analysis, writing—original draft, and writing—review and editing. All authors contributed to the article and approved the submitted version.
