# Supplementary material for: Empirically validated theoretical analysis of visual-spatial perception under change of nervous system arousal
Source: Front Comput Neurosci. 2023 May 12;17:1136985. doi: 10.3389/fncom.2023.1136985 (PMC10213702; doi:10.3389/fncom.2023.1136985)
Supplement: Supplementary file 2 [file Data_Sheet_1.pdf]

Supplementary Material

Empirically Validated Theoretical Analysis of Visual-Spatial Perception under change of Nervous System Arousal

Pratik Purohit [1], Prasun Dutta [2], Prasun K. Roy [1,3,*]

[1]School of Biomedical Engineering, Indian Institute of Technology (BHU), Varanasi, India.

[2]Department of Physics, Indian Institute of Technology (BHU), Varanasi, India.

[3]Department of Life Sciences, Shiv Nadar University (SNU), Greater Noida, India.

*** Correspondence:** Prasun K. Roy: prasun.roy@snu.edu.in

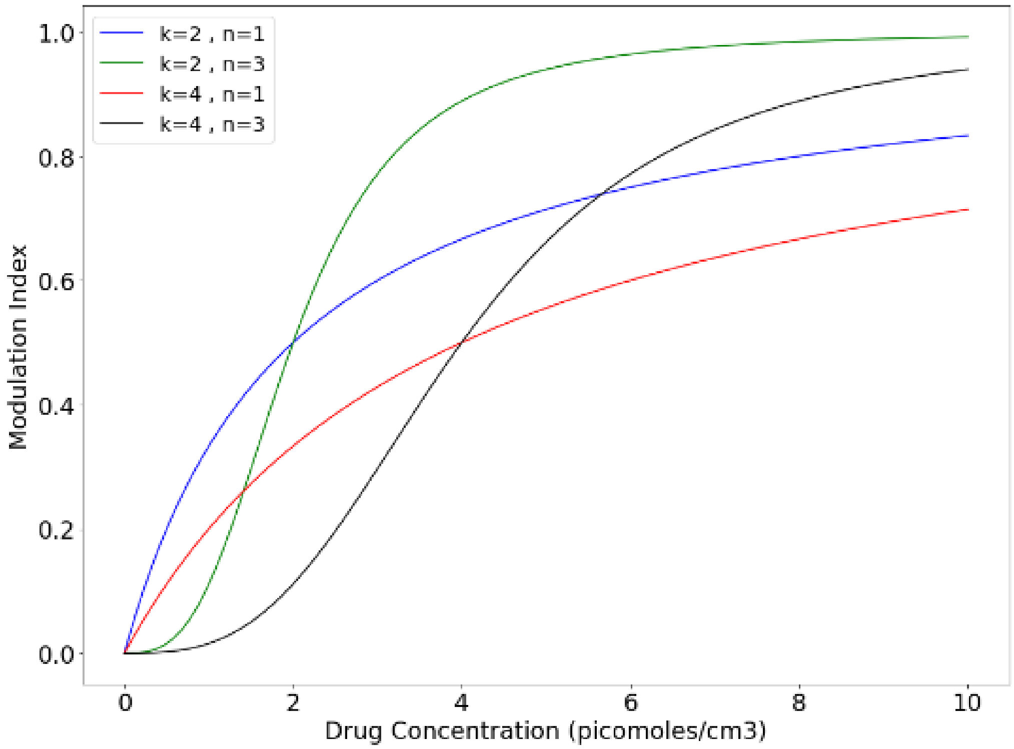

Figure S1: Variation in modulation index (M) as drug concentration (C) changes, with different values of the **n** (Hill coefficient) and **k** (Half-effective drug concentration) for illustrative purposes.

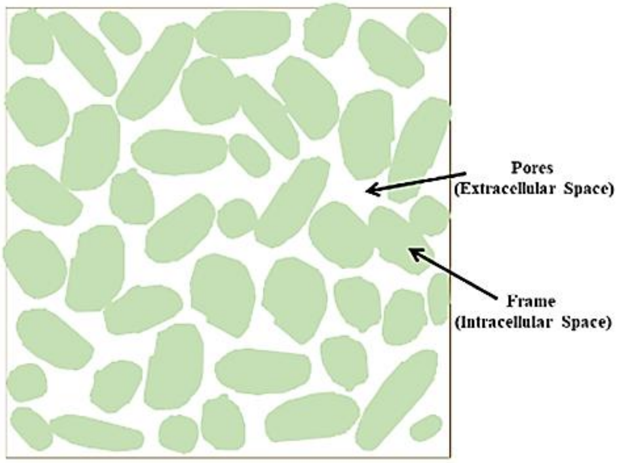

Figure S2: Simplified two-dimensional cross-sectional representation of the brain as a porous medium.

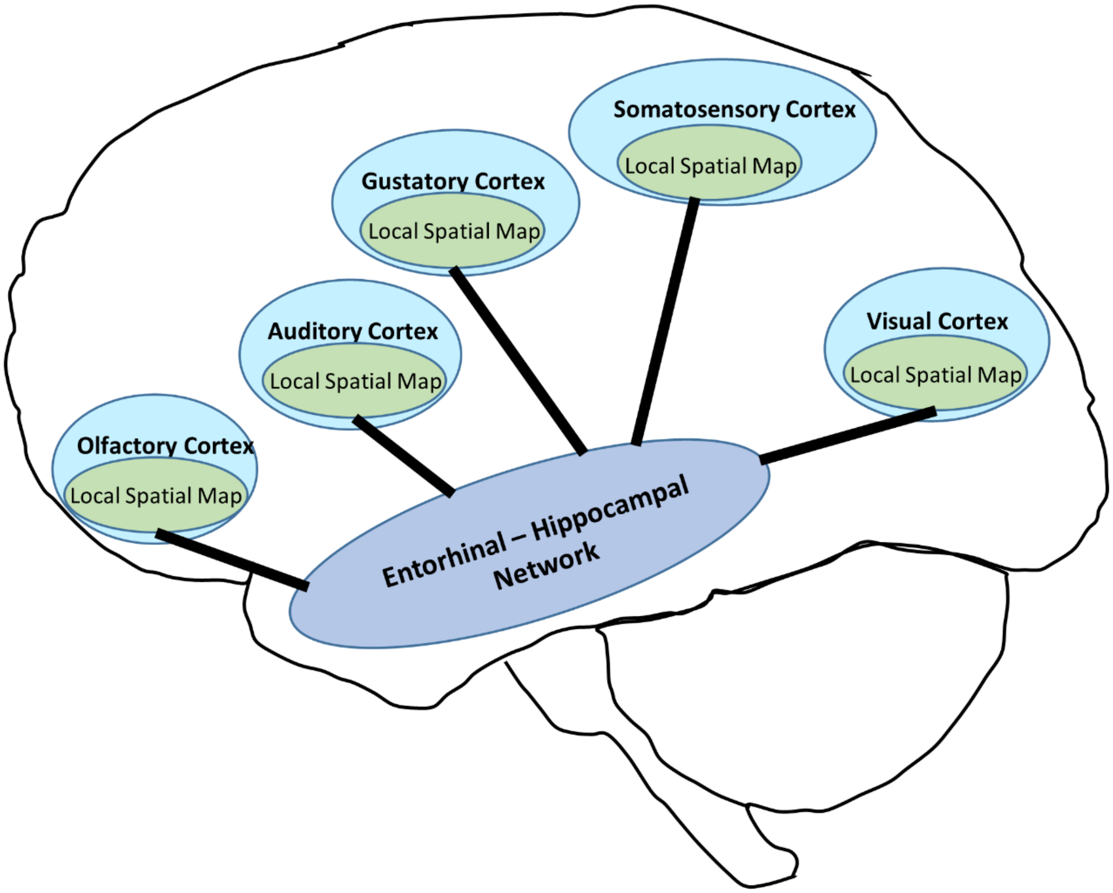

Figure S3: Conceptual interconnection complex between the Entorhinal-Hippocampal network and the various local spatial maps at different sensory cortices (e.g., visual, auditory, olfactory, or somatosensory cortices), each sensory cortex is responsible for the metric-oriented representation of the perceived sensory space in that cortex.

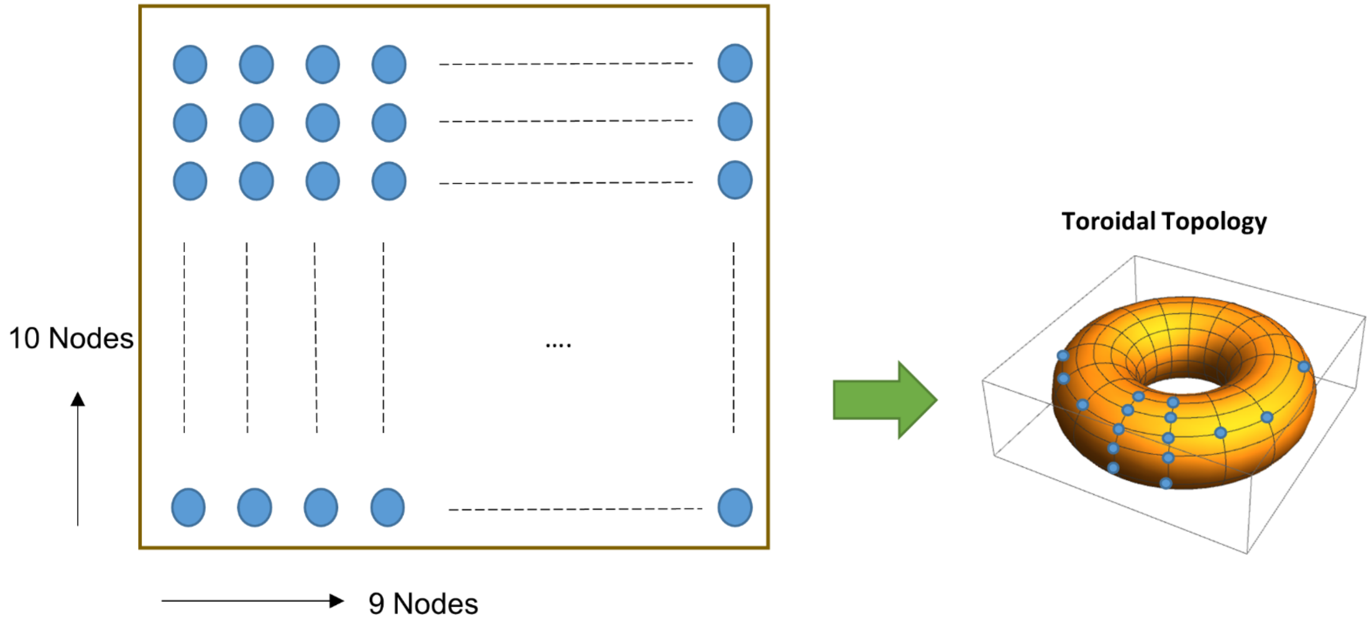

Figure S4: The arrangement of the nodes in the neural network model of the grid cells.

Section-S1 Mathematical Formulation of Modulation Index (Eq. 6) of main text:

Let us assume that the *n* molecules of the drugs can modulate the activity of one neuron, since this is a chemical process, Equations (1a) and (1b) below represent this process in terms of a chemical reaction. The terms k_f and k_r are the forward and reverse reaction rate constants, respectively. To paraphrase, a neuron has multiple binding sites for the drug molecules, out of which binding of drug molecules at *n* sites will cause the alteration in the activity of that neuron.

The forward reaction is:

$$\text{neuron} + n\ (\text{drug molecule}) \overset{k_f}{\rightarrow} \text{neuron-drug molecule complex} \tag{1a}$$

The reverse reaction is:

$$\text{neuron-drug molecule complex} \overset{k_r}{\rightarrow} \text{neuron} + n\ (\text{drug molecule}) \tag{1b}$$

Let us denote neuron by **N**, drug molecule by **C**, and neuron-drug molecule complex by **NC**. Here, **N** is a neuron whose activity is unaffected, while **NC** is a neuron whose activity is modulated by the drug action. Then Equations (1a) and (1b) become as follows:

$$\mathbf{N} + \text{n}\,(\mathbf{C}) \overset{k_f}{\rightarrow} \mathbf{NC} \tag{2a}$$

$$\mathbf{NC} \overset{k_r}{\rightarrow} \mathbf{N} + n\,(\mathbf{C}) \tag{2b}$$

Using mass-action law (Érdi and Tóth, 1989):

$$\frac{d[\mathrm{C}]}{dt} = -k_f.[N].[\mathrm{C}]^n + k_r.[N\mathrm{C}] \tag{3a}$$

$$\frac{d[N]}{dt} = -k_f.[N].[\mathrm{C}]^n + k_r.[N\mathrm{C}] \tag{3b}$$

$$\frac{d[N\mathrm{C}]}{dt} = k_f.[N].[\mathrm{C}]^n - k_r.[N\mathrm{C}] \tag{3c}$$

At equilibrium condition:

$$\frac{d[C]}{dt} = 0$$

Then, from Equation (3a):

$$k_f.[N].[\mathrm{C}]^n = k_r.[N\mathrm{C}]$$

$$[N\mathrm{C}] = \left(\frac{k_f}{k_r}\right).[N].[\mathrm{C}]^n \tag{4}$$

Adding [N] on both sides of Equation (4):

$$[N\mathrm{C}] + [N] = \left(\frac{k_f}{k_r}\right).[N].[\mathrm{C}]^n + [N] \tag{5}$$

Dividing Equation (4) by Equation (5) yields the following:

$$\frac{[N\mathrm{C}]}{[N\mathrm{C}] + [N]} = \frac{\left(\frac{k_f}{k_r}\right).[\mathrm{C}]^n}{\left(\frac{k_f}{k_r}\right).[\mathrm{C}]^n + 1}$$

$$\frac{[NC]}{[NC] + [N]} = \frac{1}{1 + \frac{k_r}{k_f [C]^n}} \tag{6}$$

Let $\frac{k_r}{k_f} = k^n$, then Equation (6) becomes:

$$\frac{[NC]}{[NC] + [N]} = \frac{1}{1 + \frac{\mathrm{k}^{\mathrm{n}}}{[C]^n}} \tag{7}$$

Now, we define the modulation index (M) as the ratio of the "number of neurons whose activity is influenced by the drug-induced nervous system activation" and "the maximum number of the neurons available for the drug action" (i.e., M can be described as the relative activation parameter of neurons, namely the fraction of the neurons that are influenced).

We can express the modulation index (M) in mathematical form, as follows:

$$M = \frac{[NC]}{[NC] + [N]} \tag{8}$$

Thereby, from Equations (7) and (8):

$$M = \frac{1}{1 + \left(\frac{\mathrm{k}}{C}\right)^n} \tag{9}$$

Let us put M=1/2 into Equation (9), then:

$$\left(\frac{\mathrm{k}}{C}\right)^n = 1$$

Since n ≠ 0, therefore k = C.

Hence the numerical value of k equals the drug concentration required to produce M=0.5 or 50 percent modulation level compared to the maximum modulation of the visual spatial perception.

Section-S2 Visual Space under Hyper-activation:

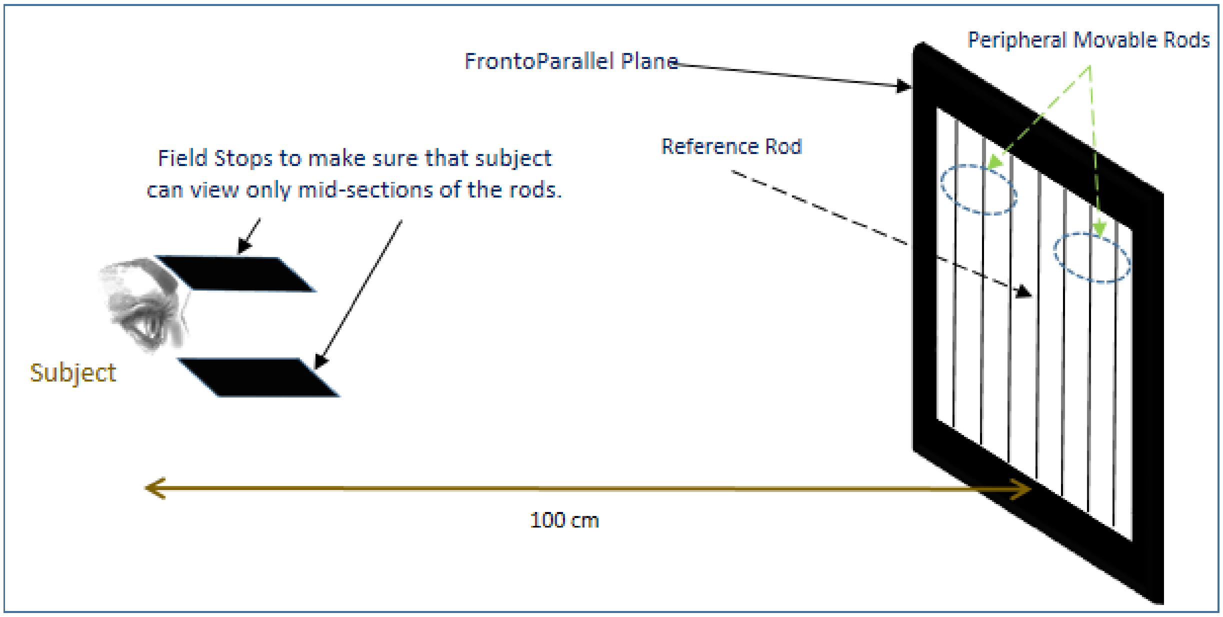

Figure S5: Experimental setup to measure the geometry of the perceived vertical fronto-parallel plane: Vertical central reference rod was placed one meter away from the subject. To avoid visual cues, the subject can see only mid-sections of the vertical peripheral rods and reference rod. The subject was instructed to move the peripheral vertical rods (towards or away from him) so that he/she could perceive all the rods in the same fronto-parallel plane.

Change in visual-spatial perception due to Psilocybin-induced hyper-activation was observed in the 16 subjects (six females and ten males, Median Age: 23.5 years). Six vertical rods, three on either side of a central vertical reference rod, were placed in the visual field at 4, 8, and 12 degrees from the pupillary sagittal plane (Supplementary Figure S5). A reference rod was placed one meter away from the subjects. Subjects could see only the middle segments of the rods to suppress any visual cues. They were told to arrange vertical rods in the frontal plane parallel to their face by giving commands to the experimenter to adjust the rods, while keeping their chin and forehead stationary. After the rearrangement of the rods in the parallel frontal plane, the positions of the rods were noted. This study procedure was repeated at 0 (just before ingestion), 90, 180, and 270 Minutes after oral ingestion (psilocybin ingestion: 160 micrograms per kg of body weight). This study method was developed by Fischer et al. (Fischer et al., 1970), who performed the experiment, and we have utilized this empirical data on the transformation of the apparent frontal plane under the effect of the agent, as reported in that paper, so as to do our further analysis, formulation and computation that we present in the "Results" section in the main text.

Section-S3   Spatial distortion threshold under hyper-activation:

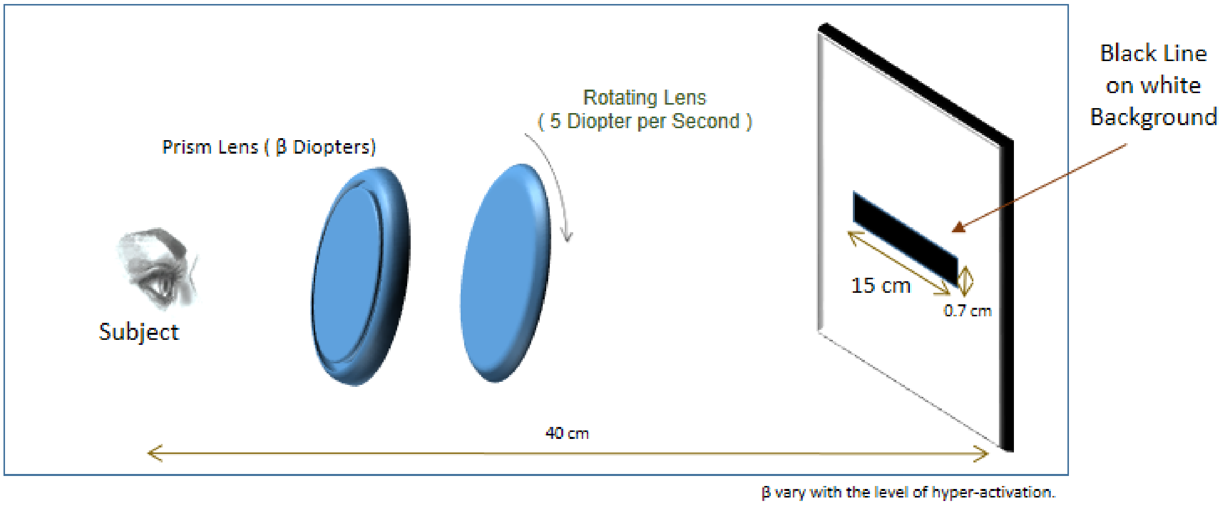

Figure S6: Experimental setup to measure the spatial distortion threshold using rotating prism lens: Rotating prism lens introduces the spatial distortion of the thick black horizontal line. The subjects view this black line through two prism lenses. The power of the second lens is kept fixed, while the power of the first lens is increased until the subject can perceive a just noticeable bending distortion in the black line. The spatial distortion threshold is measured by the minimum optical power of the first lens that is required for the subject to perceive the bending distortion.

The spatial distortion threshold in the 15 normal subjects was assessed by finding prism power required for just noticeable distortion of the horizontal 15×0.7 centimeters black line situated at 40 centimeters away from the subject (Supplementary Figure S6). Subjects viewed a black line through a rotating prism (5 prism diopters per second) and a stationary prism. The prism power of a stationary prism was gradually increased until the subject perceived the just noticeable distortion. This procedure was performed at 0 Min (before ingestion), 60, 110, and 280 minutes after oral ingestion of the amount mentioned above of the tryptamine-derivative agent (psilocybin). To paraphrase, the study procedure was formulated by Hill and Fischer (Hill et al., 1969), who performed the experiment, and we have adopted this empirical data on spatial distortion threshold under the effect of the agent as described in that paper, thereby enabling us to develop our original analysis and modeling that we show in the "Results" section in the text.

Section-S4   Spatial perception under hypo-activation:

The method in the previous section was adapted for the hypoactivation induced by oral ingestion of 50 milligrams of the phenothiazine-derivative agent (chlorpromazine). The procedure was performed at 0 minutes (before ingestion), 210, and 450 minutes after the ingestion. The spatial distortion threshold for 15 normal subjects was measured using the same experimental setup described in section S2 (figure S5 of the supplementary material). Accordingly, the experimental methodology was undertaken by Hill and Fischer (Hill et al., 1969), and we have adopted this data on spatial distortion threshold under the effect of the agent as delineated therein, so as to build up our further analysis and formulation that we furnish in the "Results" section in the main text.

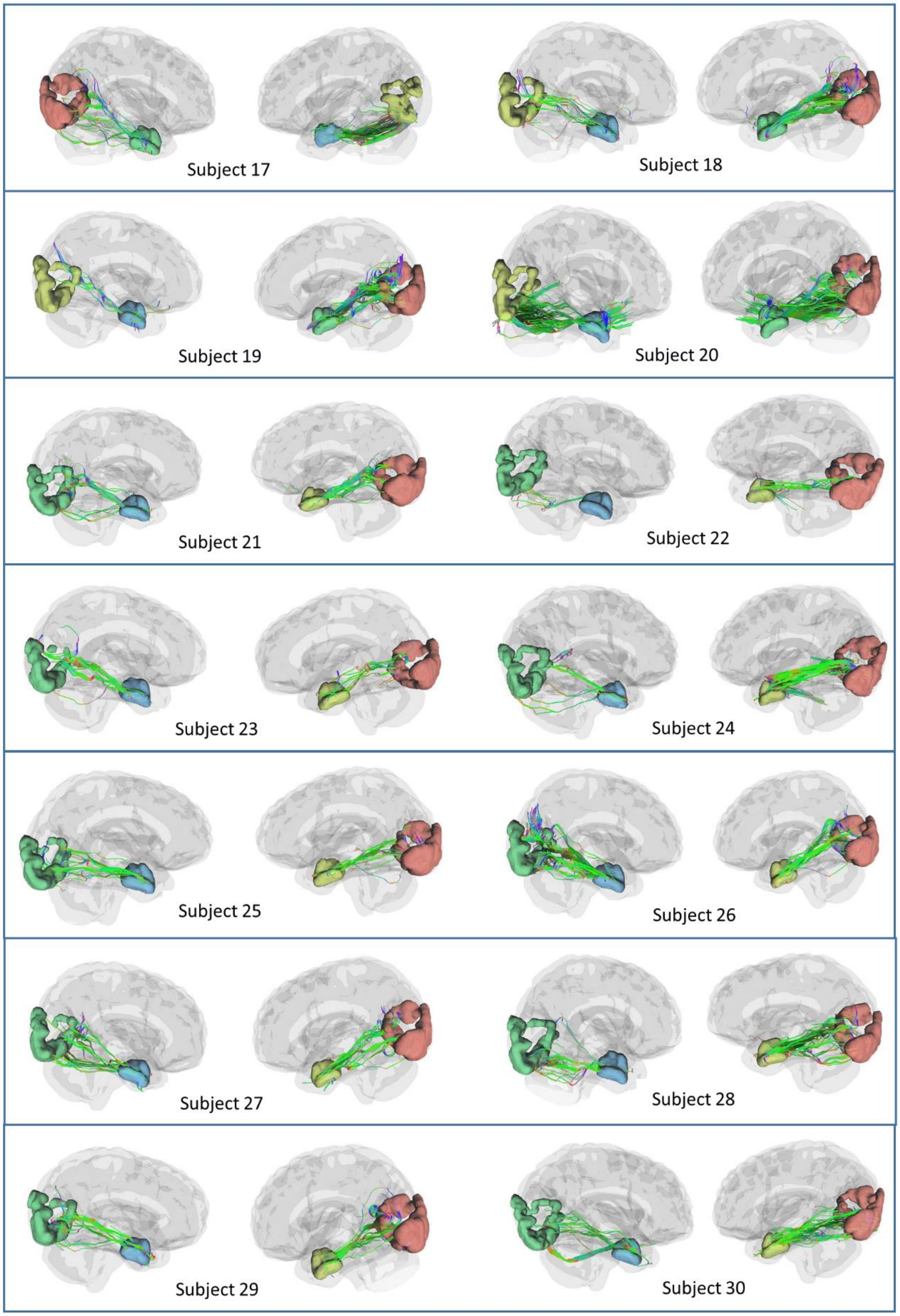

Figure S7: Anatomical connectivity (neural tracts) between the entorhinal cortex and visual cortex (area V2), obtained from MRI-tractography analysis of the fourteen subjects (Subject 17 to 30).

Section-S5 Tractography experiment using a 7 Tesla diffusion MRI scan:

Scans were acquired on a 7T Siemens MAGNETOM scanner at Maastricht University, Netherlands (Gulban et al., 2019). Approval was given by Ethics Committee of the Faculty for Psychology and Neuroscience at Maastricht University (reference number: ERCPN167_09_05_2016), and informed consent was obtained. Diffusion-weighted MRI images were scanned using multi-band diffusion-weighted spinecho EPI protocol with following parameters: b-values = 1000, 2000 and 3000 s/mm2, FOV = 200 x 200 mm with partial Fourier 6/8, 132 slices , 1.05 mm isotropic voxel size, TR = 7080 ms, TE = 75.6 ms, 66 directions and 11 additional b = 0 volumes for every b-value (Gulban et al., 2019). Susceptibility artifact was estimated using reversed phase-encoding b0 by TOPUP from the Tiny FSL package (http://github.com/frankyeh/TinyFSL), a re-compiled version of FSL TOPUP (FMRIB, Oxford) with multi-thread support. FSL eddy was used to correct for eddy current distortion. After preprocessing the MRI image, we used DSI Studio software (http://dsistudio.labsolver.org) for deterministic tractography using the diffusion tensor imaging technique (Basser et al., 1994). We used Brainnetome Atlas to locate the region of interest (ROI) (Fan et al., 2016). The tracking parameter was fractional anisotropy threshold 0.1784, angular threshold 65 degrees, step size 0.1mm, and 1000000 seeds. We performed this analysis pipeline for one normal subject (Gender: Female, Age: 27 years).

We performed the tractography between the area V2 and entorhinal cortex. The results are shown in the Figure S8, below which shows the anatomical connectivity between entorhinal cortex and area V2.

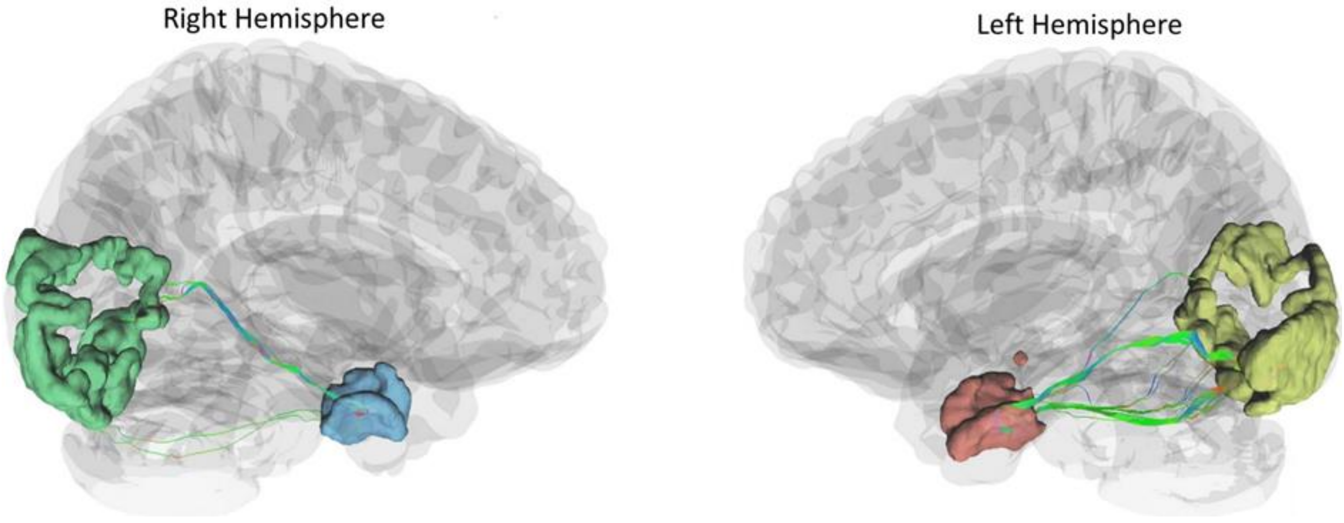

Figure S8: Anatomical connectivity (neural tracts) between the entorhinal cortex and visual cortex (area V2), obtained from MRI-tractography analysis of the a 7 Tesla diffusion MRI scan. *(cognitively normal, female, 27 years)*

References:

Basser, P. J., Mattiello, J., and Lebihan, D. (1994). Estimation of the Effective Self-Diffusion Tensor from the NMR Spin Echo. *J. Magn. Reson. Ser. B* 103, 247–254. doi:10.1006/jmrb.1994.1037.

Érdi, P., and Tóth, J. (János) (1989). Mathematical models of chemical reactions : theory and applications of deterministic and stochastic models. 259.

Fan, L., Li, H., Zhuo, J., Zhang, Y., Wang, J., Chen, L., et al. (2016). The Human Brainnetome Atlas: A New Brain Atlas Based on Connectional Architecture. *Cereb. Cortex* 26, 3508–3526. doi:10.1093/cercor/bhw157.

Fischer, R., Hill, R., Thatcher, K., and Scheib, J. (1970). Psilocybin-induced contraction of nearby visual space. *Agents Actions* 1, 190–197. doi:10.1007/BF01965761.

Gulban, O. F., Sitek, K. R., Ghosh, S. S., Moerel, M., and Martino, F. De (2019). “Auditory localization with 7T fMRI.” doi:10.18112/openneuro.ds001942.v1.2.0.

Hill, R. M., Fischer, R., and Warshay, D. (1969). Effects of excitatory and tranquilizing drugs on visual perception. spatial distortion thresholds. *Exp. 1969 252* 25, 171–172. doi:10.1007/BF01899105.
